# Long-Term Outcomes and Recovery Trajectories in Out-of-Hospital Cardiac Arrest: A 2-Year Follow-Up of the Randomized Clinical TTM2 Trial

**DOI:** 10.1001/jamaneurol.2025.5614

**Published:** 2026-02-16

**Authors:** Malin Hultgren, Erik Blennow Nordström, Susann Ullén, Niklas Nielsen, Josef Dankiewicz, Janus Christian Jakobsen, Katarina Heimburg, Marion Moseby-Knappe, Jan Bělohlávek, Mattias Bohm, Alain Cariou, Glenn Eastwood, Hans Friberg, Anders M. Grejs, Naomi Hammond, Matthias Hänggi, Juraj Hrečko, Manuela Iten, Thomas R. Keeble, Christoph Leithner, Helena Levin, Marco Mion, Christian Rylander, Claudia Schrag, Matthew Thomas, Matt P. Wise, Paul Young, Tobias Cronberg, Gisela Lilja

**Affiliations:** 1 Neurology, Department of Clinical Sciences, Lund University, Lund, Sweden; 2 Department of Rehabilitation Medicine, Skåne University Hospital, Lund, Sweden; 3 Clinical Studies Sweden – Forum South, Skåne University Hospital, Lund, Sweden; 4 Anesthesiology and Intensive Care, Department of Clinical Sciences, Lund University, Lund, Sweden; 5 Department of Intensive and Perioperative Care, Helsingborg Hospital, Helsingborg, Sweden; 6 Cardiology, Department of Clinical Sciences, Lund University, Lund, Sweden; 7 Department of Cardiology, Skåne University Hospital, Lund, Sweden; 8 Copenhagen Trial Unit, Centre for Clinical Intervention Research, Rigshospitalet, Copenhagen University Hospital, Copenhagen, Denmark; 9 Department of Regional Health Research, Faculty of Health Sciences, University of Southern Denmark, Odense, Denmark; 10 Department of Neurology, Skåne University Hospital, Lund, Sweden; 11 2nd Department of Internal Cardiovascular Medicine, General University Hospital and First Faculty of Medicine, Charles University, Prague, Czech Republic; 12 Institute for Heart Diseases, Wrocław Medical University, Wrocław, Poland; 13 Department of Intensive and Perioperative Care, Skåne University Hospital, Malmö, Sweden; 14 Cochin University Hospital (APHP) and Paris Cité University, Paris, France; 15 Department of Intensive Care, Austin Hospital, Melbourne, Victoria, Australia; 16 Department of Intensive Care Medicine, Aarhus University Hospital, Aarhus, Denmark; 17 Department of Clinical Medicine, Aarhus University Hospital, Aarhus, Denmark; 18 Critical Care Program, The George Institute for Global Health and University of New South Wales, Sydney, New South Wales, Australia; 19 Malcolm Fisher Department of Intensive Care, Royal North Shore Hospital, Sydney, New South Wales, Australia; 20 Institute of Intensive Care Medicine, University Hospital Zurich, Zurich, Switzerland; 21 1st Department of Internal Medicine - Cardioangiology, Medical Faculty of Charles University in Hradec Králové and University Hospital Hradec Králové, Hradec Králové, Czech Republic; 22 Department of Intensive Care Medicine, Inselspital, University Hospital of Bern, University of Bern, Bern, Switzerland; 23 Essex Cardiothoracic Centre, Mid and South Essex NHS Foundation Trust (MSEFT), Basildon, United Kingdom; 24 Anglia Ruskin University School of Medicine and Medical Technology Research Centre (MTRC), Chelmsford, United Kingdom; 25 Department of Neurology, Charité-Universitätsmedizin Berlin, Freie Universität Berlin and Humboldt-Universität zu Berlin, Berlin, Germany; 26 Department of Research, Development, Education and Innovation, Skåne University Hospital, Lund, Sweden; 27 Department of Surgical Sciences, Anaesthesiology and Intensive Care Medicine, Uppsala University, Uppsala, Sweden; 28 Intensive Care Department, Kantonsspital St Gallen, St Gallen, Switzerland; 29 Department of Intensive Care, Bristol Royal Infirmary, Bristol, United Kingdom; 30 Adult Critical Care, University Hospital of Wales, Cardiff, United Kingdom; 31 Intensive Care Unit, Medical Research Institute of New Zealand, Wellington Hospital, Wellington, New Zealand

## Abstract

**Importance:**

Guidelines for temperature control following out-of-hospital cardiac arrest (OHCA) are based on trials with end points of 180 days or fewer.

**Objectives:**

To investigate if targeted hypothermia, compared with targeted normothermia with early treatment of fever, affects functional outcome focusing on societal participation or cognitive functioning at 24 months in initially comatose OHCA survivors. An additional objective was to explore recovery trajectories up to 24 months post arrest.

**Design, Setting, and Participants:**

The randomized clinical Targeted Hypothermia vs Targeted Normothermia After OHCA (TTM2) trial (November 2017-2020) included blinded follow-up at 1, 6, and 24 months post randomization (December 2017-June 2022), with analyses performed in 2024. TTM2 was an international, multicenter study conducted at 61 hospitals in 14 countries. The study included 1861 adults with OHCA of presumed cardiac or unknown cause who were initially comatose. There were 992 survivors at 1 month, 943 at 6 months, and 835 at 24 months. Nonparticipation rates at follow-up were 44 (4%), 107 (11%), and 165 (20%), respectively.

**Intervention:**

Participants were randomized 1:1 to undergo temperature control via targeted hypothermia (33 °C) or targeted normothermia and early treatment of fever (≥37.8 °C).

**Main Outcomes and Measures:**

The functional outcome, including societal participation, was assessed using the Glasgow Outcome Scale-Extended (GOSE). Cognitive function was assessed using the Montreal Cognitive Assessment (MoCA) and the Symbol Digit Modalities Test (SDMT).

**Results:**

Of the participants who were followed up, 84% were male, with a mean (SD) age of 60 (14) years, and clinical variables were similar between the hypothermia and normothermia temperature groups. No significant differences were found between temperature groups regarding societal participation (GOSE: odds ratio, 0.97 [95% CI, 0.72-1.30]) or cognitive function (MoCA: mean difference, −0.02 [95% CI, −0.67 to 0.63]; SDMT: mean difference, −0.09 [95% CI, −0.33 to 0.16]) at 24 months. Improvement for GOSE was significant within the first 6 months (1 to 6 months: n = 1707 [95% CI, −2.00 to −1.50]; *P* < .001; 6 to 24 months: n = 1606 [95% CI, −0.50 to <0.001]; *P* = .10). Intraindividual improvement and decline corresponding to thresholds for minimal important differences were observed for societal participation and cognitive function up to 24 months.

**Conclusions and Relevance:**

Targeted hypothermia, compared with targeted normothermia, did not affect societal participation or cognitive function at 24 months, suggesting no longer-term effect of hypothermia for the explored outcomes. The intraindividual changes observed indicate variability in recovery.

**Trial Registration:**

ClinicalTrials.gov Identifier: NCT02908308

## Key Points

**Question:**

Does hypothermia after out-of-hospital cardiac arrest affect societal participation or cognitive functioning at 24 months post arrest, and how do these outcomes evolve over time?

**Findings:**

This follow-up of the randomized clinical Targeted Hypothermia vs Targeted Normothermia After Out-of-Hospital Cardiac Arrest trial found no significant differences in societal participation or cognitive functioning between targeted hypothermia and normothermia at 24 months. Overall recovery was limited beyond 6 months.

**Meaning:**

Targeted hypothermia compared with normothermia did not affect outcomes 24 months post arrest, suggesting no longer-term effect of hypothermia for the explored outcomes; 6 months may suffice as an end point when assessing functional or cognitive outcomes after out-of-hospital cardiac arrest.

## Teaser

This follow-up study examines the effect of hypothermia vs normothermia with early treatment of fever on long-term cognitive and functional outcomes in survivors of out-of-hospital cardiac arrest.

## Introduction

Out-of-hospital cardiac arrest (OHCA) may result in post–cardiac arrest brain injury (PCABI), which often manifests as coma following return of spontaneous circulation.^1^ Among OHCA survivors with PCABI, a majority appear to regain good functional and neurological outcomes when assessed by general outcome scales, such as the modified Rankin Scale and the Cerebral Performance Category Scale.^2,3,4,5^ Studies focusing on detailed outcomes after OHCA show that cognitive impairments—particularly those affecting memory, attention, and processing speed—are common.^6^ Cognitive function is linked to daily functioning, societal participation, and return to work.^7,8,9^ Smaller studies have evaluated functional and cognitive outcomes over time in populations with varying degrees of PCABI. The largest longitudinal follow-up study^10^ reported improved functional outcome for up to 18 months in OHCA survivors who awoke within 2 weeks of arrest. Previous studies on cognitive recovery in OHCA populations indicate that recovery stagnates within a few months.^8,11,12^

Targeted hypothermia was recommended after OHCA as a neuroprotective strategy for adults who remain comatose after return of spontaneous circulation.^13^ Current guidelines recommend active prevention of fever in patients who are comatose with OHCA. However, this recommendation is based on low-certainty evidence from trials with end points of 180 days or fewer.^14^

The randomized clinical Targeted Hypothermia vs Targeted Normothermia After OHCA (TTM2) trial^15^ found no difference between the 2 temperature groups (hypothermia and normothermia) regarding death from any cause or poor functional outcome at 6 months.^15^ An in-depth analysis of the 6-month outcomes of societal participation and cognitive function found no difference between temperature groups, although limitations in societal participation and cognitive impairment were common in both groups.^16^

This predefined, long-term follow-up of the TTM2 trial aimed to investigate if targeted hypothermia, compared with targeted normothermia with early treatment of fever, affects functional outcome focusing on societal participation or cognitive functioning at 24 months in initially comatose OHCA survivors. An additional aim was to explore recovery trajectories up to 24 months post arrest.

## Methods

### Design, Setting, and Participants

This article is presented according to the Consolidated Standards of Reporting Trials (CONSORT) extension guidelines.^17^

The TTM2 trial design, statistical analysis plan, and results from the main trial and 6-month follow-up were previously published (NCT02908308).^15,16,18,19,20^ The TTM2 trial enrolled adult (aged ≥18 years) patients with OHCA due to a presumed cardiac or unknown cause who were initially comatose, at 61 sites in 14 countries from November 2017 to January 2020. Patients were randomly assigned in a 1:1 ratio to undergo temperature control with hypothermia (33 °C) or targeted normothermia and early treatment of fever if the threshold of 37.8 °C or higher was met.

### Follow-Up Procedure and Data Collection

Trained, blinded assessors followed a written manual to conduct follow-up at 1, 6, and 24 months post randomization, from December 2017 to June 2022. Follow-up consisted of a telephone interview at 1 month and clinical follow-ups at 6 and 24 months, with the option of telephone or proxy interviews, if needed. Authorized interpreters were used when necessary. To minimize missing data due to the COVID-19 pandemic, it was possible to extend the 24-month follow-up to 36 months. Outcome assessors were occupational therapists, research nurses, psychologists, physiotherapists, or physicians. A central team provided support and performed data monitoring during the trial to prevent missing data and increase data quality.

Data regarding demographics, resuscitation, and intensive care were collected during the initial hospital stay. Characteristics collected at follow-up are listed in the follow-up protocol.^19^

### Ethical Considerations and Consent

The TTM2 trial complied with the 2013 version of the Declaration of Helsinki.^21^ Research protocols were approved by the Swedish Ethical Review Authority (2015/228) and ethical committees in all participating countries. Written informed consent was obtained from all participants who regained mental capacity.

### Outcome Assessments

The functional outcome was assessed at 1, 6, and 24 months and cognitive function at 6 and 24 months. Details of the assessments used are reported in the published protocol.^19^ Where possible, participants lost to follow-up were assigned an overall good (independent in basic activities) or poor (dependent in basic activities) outcome. This dichotomized functional outcome was informed by all available sources, including medical records and contact with health care professionals or relatives.

#### Functional Outcome Focusing on Societal Participation

The functional outcome was assessed using the clinician-reported Glasgow Outcome Scale-Extended (GOSE), an ordinal scale ranging from 1 (death) to 8 (full recovery).^22^ The assessment included a structured interview with good interrater agreement through face-to-face and telephone administration^23^ and has been used in OHCA populations.^10,24^ A GOSE score of 6 or lower indicates limitations in societal participation.^19^ Information on occupational status and return to work was used as an additional measure of societal participation, with data collected at 6 and 24 months.

#### Cognitive Functioning

Global cognitive functioning was assessed using the Montreal Cognitive Assessment (MoCA) version 7.1,^25^ the recommended assessment for cognitive impairment screening after OHCA.^14^ The MoCA score ranges from 0 to 30, with higher scores indicating better cognitive performance and a score of less than 26 indicating cognitive impairment.^25,26^ For telephone interviews, the telephone MoCA (T-MoCA; scores range from 0-22) was used, where total scores less than 19 indicate cognitive impairment.^27,28^ T-MoCA scores were converted to a 30-point MoCA scale (MoCA-30).^27^ Participants with less than 12 years of education received 1 additional point.

The Symbol Digit Modalities Test (SDMT) assessed mental processing speed and attention. The oral version was the standard, with the written version used for participants with a speech or language barrier. A face-to-face follow-up was required. Raw scores (0-110) were transformed to an age-, education-, and version-adjusted *z* score. A *z* score of −1 or less SD was used to indicate cognitive impairment.^29^ The combined use of MoCA and SDMT is suggested to improve the sensitivity for detecting cognitive impairment post OHCA and is recommended by current guidelines.^14,26^

### Statistical Analyses

Continuous data are presented as medians with IQRs or means with SDs. Categorical and binary data are presented as frequencies and percentages.

Sample size was based on the main TTM2 trial.^20^ No additional power calculation was done for the 24-month outcomes. To assess differences between temperature groups and explore recovery trajectories, the same prespecified minimal important differences (MIDs) as those used for the 6-month outcomes were used as thresholds.^19^ For GOSE, the MID is suggested to be 1 due to the direct clinical relevance of each change between categories.^19^ Two points were used as the MID for the MoCA-30 and a 0.2 *z* score for the SDMT.^19,30,31^

#### Temperature Group Comparisons

Analyses comparing intervention groups included functional outcome (GOSE) and cognitive functioning (MoCA-30 and SDMT). Initial analyses included all participants, to avoid survival bias, and were performed by the rank-based stratified Wilcoxon Mann-Whitney *U* test to adjust for site and coenrollment in the Targeted Therapeutic Mild Hypercapnia After Resuscitated Cardiac Arrest (TAME) trial.^32^ Deceased participants were considered to have the poorest outcome (lowest rank) in these analyses and were therefore assigned GOSE 1 (death). For MoCA and SDMT, deceased participants were assigned a score (ie, rank) lower than the lowest possible for survivors.^33^

For survivor-only analyses, mixed-effects ordinal regression for GOSE and mixed-effects linear regression for MoCA and SDMT were used. Two models were performed for each outcome, where model 1 included adjustment for site (random intercept) and coenrollment in the TAME trial. To account for the balanced allocation by the randomization process possibly being affected by survival bias, model 2 included adjustment for site (random intercept), coenrollment, age (younger than 65 years/65 years or older), sex (male/female), education (any university studies: yes/no), and prearrest Clinical Frailty Scale^34^ score (fit or prefrail, 1-4; frail, 5-9), if a covariate was not already accounted for in the scoring of the assessment.

#### Recovery Trajectories

The analyses exploring recovery trajectories were preplanned to be stratified by temperature group if significant differences between the temperature groups were found; otherwise, the OHCA survivors were planned to be analyzed as 1 group.

Unadjusted analyses were used to explore group-level differences over time. The rank-based Wilcoxon signed rank test was used for GOSE (ordinal variable) and paired *t* tests for MoCA and SDMT (continuous variables). All participants, including deceased individuals, were included for GOSE and only survivors for MoCA and SDMT. Two sensitivity analyses with survivors only for GOSE were performed using the Wilcoxon signed rank test.

Multivariable linear regression examined the effects of age, education, and sex on cognitive recovery, if not already accounted for in the scoring. Age and education were chosen due to their established association with cognitive function.^35,36,37^ Sex was included because women may have worse outcomes than men after OHCA.^38,39^ The dependent variable for the model was the δ of MoCA and SDMT, respectively (24 months compared with 6 months). Reference categories were age younger than 65 years, no university education, and male sex.

Analyses were performed during 2024. Tests were 2-sided and *P* < .05 was the threshold for statistical significance. Results are considered exploratory, with no adjustment for multiplicity. SPSS Statistics version 28.0 (IBM Corporation) and R version 4.4.2 (R Foundation) were used in statistical analyses.

## Results

Of the 1861 participants in the TTM2 trial, 992 (53%) were alive at 1 month, 943 (51%) at 6 months, and 835 (45%) at 24 months. Participation rates during follow-up were 948 of 992 (95%) at 1 month, 836 of 943 (89%) at 6 months, and 670 of 835 (80%) at 24 months (eFigure in Supplement 1). The characteristics of follow-up participants are presented in Table 1. The number of available assessments for the main outcome are presented in the eFigure in Supplement 1. The dichotomized functional outcome assigned to participants lost to follow-up was available for 82 of 83 (99%) of participants lost to follow-up at 6 months and 105 of 165 (64%) at 24 months.

**Table 1.** Participant Characteristics^a^

Variable	Follow-up, No. (%)					
	1 mo		6 mo		24 mo	
	Hypothermia (n = 458)	Normothermia (n = 490)	Hypothermia (n = 416)	Normothermia (n = 420)	Hypothermia (n = 332)	Normothermia (n = 338)
Pre-OHCA						
Age, mean (SD), y at time of cardiac arrest	60 (14)	61 (14)	60 (13)	59 (14)	61 (13)	58 (14)
University-level education^b^	NA	NA	137 (33)	130 (32)	108 (34)	110 (34)
Sex						
Male	388 (85)	405 (83)	354 (85)	346 (82)	284 (86)	283 (84)
Female	70 (15)	85 (17)	62 (15)	74 (18)	48 (14)	55 (16)
Medical history [prior to cardiac arrest]						
Charlson Comorbidity Index, median (IQR)	2 (1-3)	2 (1-3)	2 (1-3)	2 (1-3)	2 (1-3)	2 (1-3)
Prearrest frailty [CFS >4]	10 (2)	15 (3)	4 (1)	11 (3)	4 (1)	3 (1)
Diabetes	71 (15)	72 (15)	57 (14)	55 (13)	43 (13)	45 (13)
Heart failure	21 (5)	30 (6)	23 (6)	27 (7)	15 (5)	20 (6)
Hypertension with pharmacologic treatment	149 (34)	149 (32)	139 (35)	124 (31)	110 (35)	98 (30)
Known neurological disease^b^	NA	NA	33 (8)	27 (7)	24 (7)	19 (6)
Memory problems [self-reported]^b^	NA	NA	31 (8)	32 (8)	24 (7)	25 (7)
Myocardial infarction	56 (13)	73 (16)	53 (13)	62 (15)	40 (13)	49 (15)
Poor functional outcome/status [mRS 4-5]	1 (<0.1)	1 (<0.1)	0	0	0	0
OHCA resuscitation variables						
Location of cardiac arrest, at home	196 (43)	217 (44)	175 (42)	181 (43)	143 (43)	140 (45)
Bystander-witnessed arrest	425 (93)	455 (93)	383 (92)	388 (92)	308 (93)	309 (92)
First monitored rhythm, shockable	402 (88)	428 (87)	371 (89)	380 (91)	294 (89)	312 (93)
Time to sustained ROSC, median (IQR), min	20 (14-31)	20 (14-30)	20 (14-30)	20 (14-30)	20 (14-31)	20 (14-30)
Data on hospital admission						
Shock	93 (20)	103 (21)	84 (20)	85 (20)	70 (21)	65 (19)
In-hospital						
Days in hospital, median (IQR)	16 (11-25)	15 (10-26)	16 (11-25)	15 (10-24)	16 (11-24)	15 (10-24)
Days in intensive care unit, median (IQR)	6 (4-10)	5 (3-10)	6 (4-9)	5 (3-9)	6 (4-9)	5 (3-9)
At follow-up						
Days from cardiac arrest to follow-up, median (IQR)	30 (28-34)	30 (29-35)	186 (179-200)	187 (179-200)	755 (734-826)	758 (734-841)
Follow-up, face to face^c^	NA	NA	313 (75)	306 (73)	197 (59)	211 (62)
Living at home	296 (65)	312 (64)	403 (97)	384 (94)	324 (98)	324 (96)
Married/living as married^b^	NA	NA	304 (73)	305 (75)	263 (79)	256 (76)
Rehabilitation provided [self-reported]						
Cardiac rehabilitation^b^^,^^d^	NA	NA	121 (29)	111 (26)	109 (33)	109 (32)
Exercise-based cardiac rehabilitation^b^^,^^d^	NA	NA	79 (19)	88 (21)	65 (20)	70 (21)
Inpatient neurological/cognitive/brain injury rehabilitation^b^^,^^d^	NA	NA	49 (12)	50 (12)	39 (12)	34 (10)
Outpatient neurological/cognitive/brain injury rehabilitation^b^^,^^d^	NA	NA	22 (5)	29 (7)	18 (5)	22 (7)
Other^b^^,^^d^	NA	NA	24 (5)	18 (4)	10 (3)	8 (2)

Abbreviations: CFS, Clinical Frailty Scale; mRS, modified Rankin Scale; NA, not applicable; OHCA, out-of-hospital cardiac arrest; ROSC, return of spontaneous circulation.

^a^ Continuous data presented as median with IQR or mean with SD. Categorical and binary data are presented as frequency with percentages.

^b^ Not included in 1-month follow-up.

^c^ Follow-up performed via telephone only at 1 month.

^d^ Categories are not mutually exclusive.

### Temperature Group Comparisons

**Figure 1.**
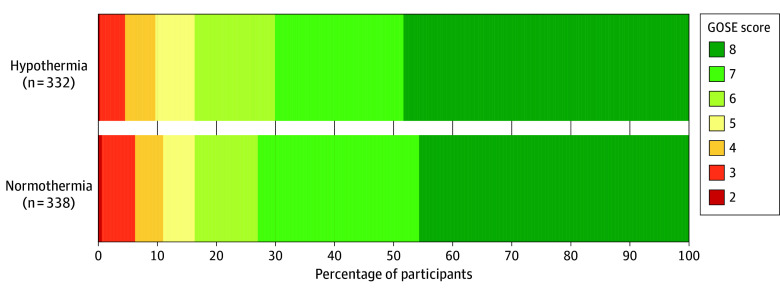
Component Bar Graph of Glasgow Outcome Scale-Extended (GOSE) Scores at 24 Months, Stratified by Temperature Group Distribution of GOSE scores of survivors who participated in the 24-month follow-up, stratified by temperature group. A GOSE score of 8 represents a full recovery and 2 represents a survivor in a nonresponsive state.

No significant difference in the functional outcome focusing on societal participation was found between temperature groups at 24 months in any of the analyses (estimate for all participants, −0.1 [95% CI, −0.03 to 0.02]; *P* = .68; adjusted odds ratio, 0.97 [95% CI, 0.72-1.30]) (eTable 1 in Supplement 1). The distribution of GOSE scores for the 24-month follow-up participants was similar between the temperature groups (Figure 1).

No significant differences between temperature groups were found for cognitive outcomes (MoCA-30: estimate for all participants, −0.02 [95% CI, −0.04 to 0.01]; *P* = .27; adjusted model 2, mean difference [MD] −0.02 [95% CI, −0.67 to 0.63]; SDMT: estimate for all participants, −0.01 [95% CI, −0.03 to 0.02]; *P* = .65; adjusted model 2: MD, −0.09 [95% CI, −0.33 to 0.16]) (eTable 1 in Supplement 1). The number of participants below the cutoff was similar between temperature groups (eTable 2 in Supplement 1).

### Recovery Trajectories

Because no differences in the analyses for temperature group comparisons were found, participants were analyzed as 1 group in the analyses of recovery trajectories. Outcomes at 6 and 24 months for all follow-up participants and participants in paired analyses are presented in Table 2. Among participants with an available GOSE score at both time points, there was a significant difference in GOSE between 1 and 6 months (n = 1707 [95% CI, −2.00 to −1.50]; *P* < .001) but not between 6 and 24 months (n = 1606 [95% CI, −0.50 to <0.001]; *P* = .10). Results were the same in the sensitivity analyses including survivors only.

**Table 2.** Functional and Cognitive Outcomes at 6 and 24 Months

Outcome	Follow-up			
	6 mo	Participants in paired analyses, 6 mo	24 mo	Participants in paired analyses, 24 mo
GOSE, No.	834	648	670	648
GOSE ≤6, No. (%)	346 (41)	259 (40)	191 (29)	183 (28)
MoCA-30, No.^a^	760	571	609	571
MoCA-30, median (IQR)^a^	26 (23 to 29)	27 (24 to 29)	26 (23 to 28)	26 (23 to 28)
MoCA-30 <26, No. (%)^a^	330 (43)	221 (39)	278 (46)	254 (44)
MoCA, No.	607	468	398	383
MoCA, median (IQR)	27 (23 to 29)	27 (24 to 29)	26 (23 to 28)	26 (24 to 29)
MoCA <26, No. (%)	248 (41)	170 (36)	157 (39)	149 (38)
T-MoCA, No.	153	103	211	188
T-MoCA median (IQR)	19 (17 to 21)	20 (18 to 21)	19 (17 to 21)	22 (17 to 21)
T-MoCA <19, No. (%)	61 (40)	35 (34)	87 (41)	74 (39)
SDMT, No.	601	356	390	356
SDMT *z* score, median (IQR)	−0.94 (−1.84 to −0.15)	−0.88 (−1.70 to −0.07)	−0.72 (−1.60 to −0.11)	−0.67 (−1.55 to −0.10)
SDMT *z* score, mean (SD)	−1.05 (1.38)	−0.97 (1.30)	−0.87 (1.34)	−0.85 (1.26)
SDMT SD ≤−1, No. (%)	286 (48)	159 (45)	164 (42)	147 (41)
SDMT SD ≤−1.5, No. (%)	193 (32)	102 (29)	107 (27)	94 (26)
MoCA-30 and SDMT, No.	600	355	390	355
MoCA-30 or SDMT under cutoff, No. (%)^a^	353 (59)	195 (55)	221 (57)	201 (57)
MoCA-30 and SDMT under cutoff, No. (%)^a^	176 (29)	95 (27)	97 (25)	85 (24)

Abbreviations: GOSE, Glasgow Outcome Scale-Extended; MoCA, Montreal Cognitive Assessment; SDMT, Symbol Digit Modalities Test; T-MoCA, telephone MoCA.

^a^ Includes converted T-MoCA.

**Figure 2.**
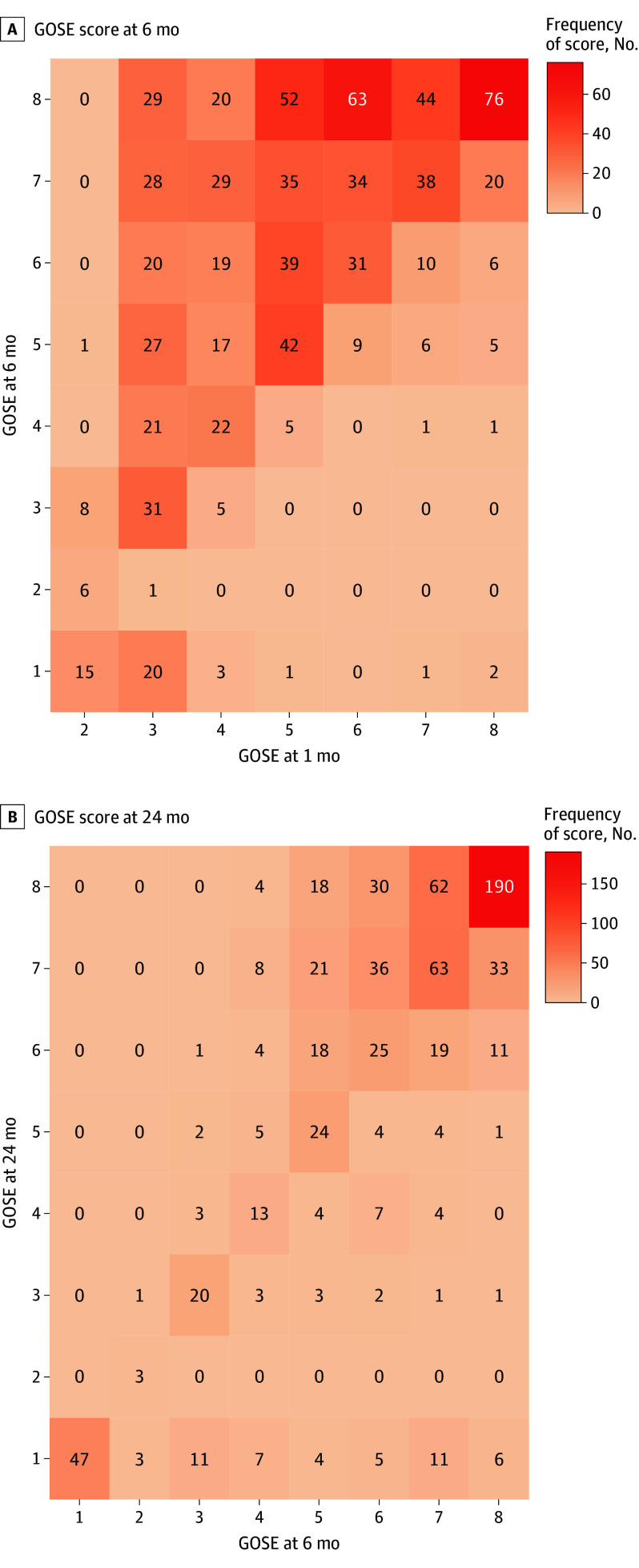
Heat Map of Glasgow Outcome Scale-Extended (GOSE) Trajectories Between 1 and 24 Months Trajectories of GOSE scores between 1 to 6 months (n = 843) and 6 to 24 months (n = 740). A GOSE score of 8 represents a full recovery and 1 represents death. The population presented is survivors alive at 1 month who had available GOSE scores.

**Figure 3.**
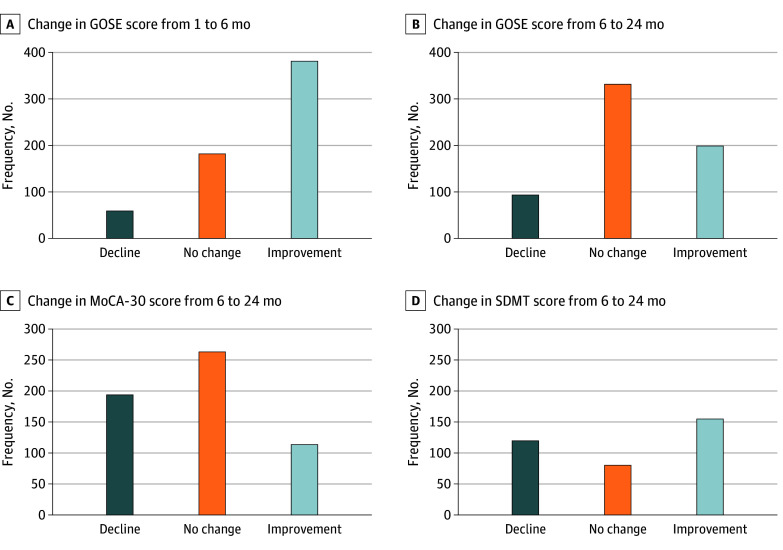
Bar Graph of Changes in Outcome Assessments Intraindividual change in scores between GOSE, MoCA-30, and SDMT, categorized by the minimal important difference (1 category for GOSE, 2 points for MoCA-30, and 0.2 *z* score for SDMT). A and B (n = 625), 24-Month participants with available GOSE score at all time points. C and D, Participants who had data on MoCA-30 (n = 571) and SDMT (n = 356) at both 6 and 24 months. MoCA-30 includes converted telephone MoCA. A sensitivity analysis where *z* scores for SDMT at 24 months were calculated using the ages at 6 months to account for possible aging into another age bracket for the normative values yielded similar results. GOSE indicates Glasgow Outcome Scale-Extended; MoCA, Montreal Cognitive Assessment; SDMT, Symbol Digit Modalities Test.

Intraindividual improvement and decline in GOSE scores that reached the MID threshold were observed beyond 6 months (Figures 2 and 3). Participants who improved beyond 6 months for GOSE were generally younger (mean [SD] age, 58 [13] years vs 65 [12] years), had higher rates of university education (36% vs 27%), and higher participation rates in cardiac rehabilitation at 6 months (34% vs 22%) compared with those who declined (eTable 3 in Supplement 1).

For follow-up participants with GOSE across all time points (n = 625), limitations in societal participation (GOSE ≤6) decreased from 453 (72%) at 1 month to 250 (40%) at 6 months and 178 (28%) at 24 months. Participants lost to follow-up had higher rates of poor outcomes compared with follow-up participants at both 6 and 24 months (6 months, 21 of 82 [26%] vs 56 of 836 [7%]; 24 months, 26 of 105 [25%] vs 40 of 670 [6%]).

Results on return to work are presented in eTable 4 in Supplement 1. Of the participants working prearrest with information on occupational status at 24 months, 249 of 352 (71%) had returned to work. Participants working full time increased from 182 of 438 (42%) to 197 of 352 (56%) between 6 and 24 months. Sick leave decreased from 95 of 438 (22%) to 11 of 352 (3%).

Cognitive outcomes for all follow-up participants and participants in paired analyses are presented in Table 2. For participants with cognitive assessments at both time points, there was a significant difference for MoCA-30 (n = 571; MD, 0.61 [95% CI, 0.37-0.85]; *P* < .001) and SDMT (n = 356; MD, −0.11 [95% CI, −0.21 to −0.02]; *P* = .02) between 6 and 24 months; the MDs were below the MID thresholds.

Intraindividual improvement and decline in MoCA-30 and SDMT scores that reached the MID thresholds were observed beyond 6 months (Figure 3). Like the GOSE, participants with an improvement beyond 6 months had higher rates of university education (42% vs 25%) compared with those who declined, but mean (SD) age (58 [15] years vs 60 [13] years) and participation in cardiac rehabilitation (25% vs 27%) were similar (eTable 3 in Supplement 1).

Among the participants with cognitive assessments at both 6 and 24 months, the number of participants scoring below the cutoff indicative of cognitive impairment was 221 of 571 (40%) at 6 months and 254 of 571 (44%) at 24 months on the MoCA-30 and 159 of 356 (45%) vs 147 of 356 (41%) for SDMT. Item-specific results of the MoCA-30 are shown in eTable 5 in Supplement 1. Results from the linear regressions are presented in eTable 6 in Supplement 1.

## Discussion

In this long-term follow-up of the TTM2 trial, it was found that targeted hypothermia, compared with targeted normothermia and early treatment of fever, did not affect societal participation or cognitive functioning in initially comatose OHCA survivors at 24 months post arrest. These findings are consistent with current literature and guidelines on temperature control but extend the knowledge beyond 6 months.^14,40^ Significant improvement in societal participation within the first 6 months was found, although overall recovery beyond 6 months was limited for both societal participation and cognitive function.

That the majority of recovery in societal participation was observed within the first 6 months supports the results from a previous trial.^10^ At 24 months, 28% of participants reported limitations in societal participation, lower than previously reported at 12 (44%) and 18 (35%) months.^10,24^ The international design of the study may offer a broader perspective on long-term functional outcomes because prior studies were nation-specific and outcomes may vary by region.^10,24,41^ Multiple points of follow-up meant participants had opportunities to be referred to further care, potentially improving outcomes.

The rates of cognitive impairment remained largely unchanged over time and the limited recovery in cognitive function is similar to previous literature on detailed cognitive recovery after OHCA.^8,11,12^ Recovery trajectories differed on the MoCA-30 and SDMT, with more individuals improving on the SDMT. This difference may be due to selection bias, as the SDMT required a face-to-face follow-up with higher levels of missing data. Detailed studies exploring domain-specific cognitive recovery post OHCA may also provide further insight. Overall, this study supports previous evidence that 40% to 50% of OHCA survivors have long-term cognitive impairments, although detailed comparisons are difficult due to large heterogeneities in cognitive outcome reporting.^6,14^ Previous studies have reported mixed associations of older age and female sex with cognitive outcomes after cardiac arrest, which was also observed in this study.^38,39,42^

Although most participants did not improve beyond 6 months, some participants did demonstrate further improvement that met the MID thresholds. The use of an MID may strengthen clinical applicability because an MID aims to reflect the smallest change that is potentially clinically meaningful.^43^ Although MID is not without its shortcomings,^44^ study results indicate that younger participants and those with university education were more likely to improve beyond 6 months in societal participation, with university education, although not age, seemingly linked to improvements in cognitive function as well. Potential explanations for the delayed improvement in societal participation for the younger survivors may be related to different causes of OHCA or the increase in return to work rates between 6 and 24 months.^45^ Further research is needed to explore factors that may affect continued improvements in societal participation and cognitive function, including etiology and rehabilitation.

This study adds information of no long-term effect of targeted hypothermia for the explored outcomes, which is not currently represented in systematic reviews on temperature control or postresuscitation guidelines.^14,40^ The detailed analyses of the recovery trajectories of functional outcomes and cognitive function may aid in the timing of outcome reporting in future trials and guidelines because 6 months may be an adequate end point. The Core Outcome Set for Cardiac Arrest in Adults statement recommends reporting functional outcomes at hospital discharge or 30 days,^46^ while study findings indicate that this is too early to determine definitive functional outcomes. Current postresuscitation care guidelines recommend follow-up within 3 months after hospital discharge to screen for cognitive impairment post arrest.^14^ Although 3 months was not included as a time point, study findings support cognitive screening within 6 months. Studies are needed to explore recovery trajectories for other outcomes.

### Strengths and Limitations

Strengths include being the largest international, multicenter randomized clinical trial with follow-up of OHCA survivors up to 24 months, with strategies to increase interassessor reliability and data quality and minimize missing data. This study has limitations. First, loss to follow-up reached 20% at 24 months. Participants lost to follow-up had higher rates of poor outcomes, which may skew results toward more favorable outcomes, and the possibility that participants lost to follow-up may have different recovery trajectories than the ones reported cannot be excluded, although missing data are unlikely to alter conclusions. Second, only OHCA of presumed cardiac or unknown cause was included, meaning less generalizability to OHCA of other causes, such as drug use or suicide. The mainly European study population also limits generalizability to other settings, such as the US, where there is a higher incidence of emergency medical services–treated OHCA and lower national rates of bystander cardiopulmonary resuscitation.^45,47^ Third, telephone-based follow-up was more common at 24 months due to the COVID-19 pandemic, thus missing data for the SDMT increased and the MoCA and T-MoCA ratio was changed, which may affect comparisons.^27^ Fourth, due to the unexpected nature of OHCA, cognitive function was not captured at baseline.

## Conclusions

At 24 months, targeted hypothermia, compared with targeted normothermia and early treatment of fever, did not affect the functional outcome focusing on societal participation or cognitive function in initially comatose OHCA survivors. There was significant recovery in societal participation within the first 6 months but overall recovery thereafter was limited for both functional and cognitive outcomes. The intraindividual changes observed indicate variability in recovery.

## Notes

Supplement 1. eMethods 1. Sites in the TTM2 trial eMethods 2. Changes From the Published Protocol eMethods 3. R-packages Used for Statistical analyses and Figures eTable 1. Analysis Comparing Functional Outcome and Cognitive Function in Out-of-Hospital Cardiac Arrest Survivors Treated with Hypothermia versus Normothermia eTable 2. Functional and Cognitive Outcomes at 6 and 24 Months, stratified by temperature group eTable 3. Participant Characteristics Stratified by Change in Assessment Scores Between 6 & 24 Months eTable 4. Occupational status, Pre-arrest Workers eTable 5. Item-specific Results on the MoCA-30 eTable 6. Analysis Exploring Impact of Age, Education and Sex on Cognitive Outcome eFigure. CONSORT Flowchart of Participants and Loss to-follow-up eReferences

Supplement 2. Trial Protocol

Supplement 3. Data Sharing Statement

## References

[1] Sandroni C, Cronberg T, Sekhon M. Brain injury after cardiac arrest: pathophysiology, treatment, and prognosis. Intensive Care Med. 2021;47(12):1393-1414. doi:10.1007/s00134-021-06548-2 34705079 PMC8548866

[2] Edgren E, Hedstrand U, Kelsey S, Sutton-Tyrrell K, Safar P; BRCT I Study Group. Assessment of neurological prognosis in comatose survivors of cardiac arrest. Lancet. 1994;343(8905):1055-1059. doi:10.1016/S0140-6736(94)90179-1 7909098

[3] Nichol G, Guffey D, Stiell IG, ; Resuscitation Outcomes Consortium Investigators. Post-discharge outcomes after resuscitation from out-of-hospital cardiac arrest: a ROC PRIMED substudy. Resuscitation. 2015;93:74-81. doi:10.1016/j.resuscitation.2015.05.011 26025570

[4] Vaahersalo J, Hiltunen P, Tiainen M, ; FINNRESUSCI Study Group. Therapeutic hypothermia after out-of-hospital cardiac arrest in Finnish intensive care units: the FINNRESUSCI study. Intensive Care Med. 2013;39(5):826-837. doi:10.1007/s00134-013-2868-1 23417209

[5] Nielsen N, Wetterslev J, Cronberg T, ; TTM Trial Investigators. Targeted temperature management at 33°C versus 36°C after cardiac arrest. N Engl J Med. 2013;369(23):2197-2206. doi:10.1056/NEJMoa1310519 24237006

[6] Zook N, Voss S, Blennow Nordström E, . Neurocognitive function following out-of-hospital cardiac arrest: a systematic review. Resuscitation. 2022;170:238-246. doi:10.1016/j.resuscitation.2021.10.005 34648921

[7] Boyce-van der Wal LW, Volker WG, Vliet Vlieland TP, van den Heuvel DM, van Exel HJ, Goossens PH. Cognitive problems in patients in a cardiac rehabilitation program after an out-of-hospital cardiac arrest. Resuscitation. 2015;93:63-68. doi:10.1016/j.resuscitation.2015.05.029 26066808

[8] Ørbo M, Aslaksen PM, Larsby K, Schäfer C, Tande PM, Anke A. Alterations in cognitive outcome between 3 and 12 months in survivors of out-of-hospital cardiac arrest. Resuscitation. 2016;105:92-99. doi:10.1016/j.resuscitation.2016.05.017 27255953

[9] Lilja G, Nielsen N, Bro-Jeppesen J, . Return to work and participation in society after out-of-hospital cardiac arrest. Circ Cardiovasc Qual Outcomes. 2018;11(1):e003566. doi:10.1161/CIRCOUTCOMES.117.003566 29326145

[10] Peskine A, Cariou A, Hajage D, ; Hanox Study Group. Long-term disabilities of survivors of out-of-hospital cardiac arrest: the Hanox study. Chest. 2021;159(2):699-711. doi:10.1016/j.chest.2020.07.022 32702410

[11] Lim C, Verfaellie M, Schnyer D, Lafleche G, Alexander MP. Recovery, long-term cognitive outcome and quality of life following out-of-hospital cardiac arrest. J Rehabil Med. 2014;46(7):691-697. doi:10.2340/16501977-1816 24849762 PMC4111096

[12] Steinbusch CVM, van Heugten CM, Rasquin SMC, Verbunt JA, Moulaert VRM. Cognitive impairments and subjective cognitive complaints after survival of cardiac arrest: a prospective longitudinal cohort study. Resuscitation. 2017;120:132-137. doi:10.1016/j.resuscitation.2017.08.007 28818523

[13] Nolan JP, Sandroni C, Böttiger BW, . European Resuscitation Council and European Society of Intensive Care Medicine guidelines 2021: post-resuscitation care. Intensive Care Med. 2021;47(4):369-421. doi:10.1007/s00134-021-06368-4 33765189 PMC7993077

[14] Nolan JP, Sandroni C, Cariou A, . European Resuscitation Council and European Society of Intensive Care Medicine guidelines 2025: post-resuscitation care. Intensive Care Med. 2025;51(12):2213-2288. doi:10.1007/s00134-025-08117-3 41123621

[15] Dankiewicz J, Cronberg T, Lilja G, ; TTM2 Trial Investigators. Hypothermia versus normothermia after out-of-hospital cardiac arrest. N Engl J Med. 2021;384(24):2283-2294. doi:10.1056/NEJMoa2100591 34133859

[16] Lilja G, Ullén S, Dankiewicz J, . Effects of hypothermia vs normothermia on societal participation and cognitive function at 6 months in survivors after out-of-hospital cardiac arrest: a predefined analysis of the TTM2 randomized clinical trial. JAMA Neurol. 2023;80(10):1070-1079. doi:10.1001/jamaneurol.2023.2536 37548968 PMC10407762

[17] Hopewell S, Chan AW, Collins GS, . CONSORT 2025 statement: updated guideline for reporting randomized trials. JAMA. 2025;333(22):1998-2005. doi:10.1001/jama.2025.4347 40228499

[18] Dankiewicz J, Cronberg T, Lilja G, . Targeted hypothermia versus targeted normothermia after out-of-hospital cardiac arrest (TTM2): a randomized clinical trial—rationale and design. Am Heart J. 2019;217:23-31. doi:10.1016/j.ahj.2019.06.012 31473324

[19] Lilja G, Nielsen N, Ullén S, . Protocol for outcome reporting and follow-up in the Targeted Hypothermia versus Targeted Normothermia after Out-of-Hospital Cardiac Arrest trial (TTM2). Resuscitation. 2020;150:104-112. doi:10.1016/j.resuscitation.2020.03.004 32205155

[20] Jakobsen JC, Dankiewicz J, Lange T, . Targeted hypothermia versus targeted normothermia after out-of-hospital cardiac arrest: a statistical analysis plan. Trials. 2020;21(1):831. doi:10.1186/s13063-020-04654-y 33028425 PMC7542893

[21] World Medical Association. World Medical Association Declaration of Helsinki: ethical principles for medical research involving human subjects. JAMA. 2013;310(20):2191-2194. doi:10.1001/jama.2013.281053 24141714

[22] Wilson JT, Pettigrew LE, Teasdale GM; Glasgow Outcome Scale and the Extended Glasgow Outcome Scale. Structured interviews for the Glasgow Outcome Scale and the extended Glasgow Outcome Scale: guidelines for their use. J Neurotrauma. 1998;15(8):573-585. doi:10.1089/neu.1998.15.573 9726257

[23] McMillan T, Wilson L, Ponsford J, Levin H, Teasdale G, Bond M. The Glasgow Outcome Scale—40 years of application and refinement. Nat Rev Neurol. 2016;12(8):477-485. doi:10.1038/nrneurol.2016.89 27418377

[24] Smith K, Andrew E, Lijovic M, Nehme Z, Bernard S. Quality of life and functional outcomes 12 months after out-of-hospital cardiac arrest. Circulation. 2015;131(2):174-181. doi:10.1161/CIRCULATIONAHA.114.011200 25355914

[25] Nasreddine ZS, Phillips NA, Bédirian V, . The Montreal Cognitive Assessment, MoCA: a brief screening tool for mild cognitive impairment. J Am Geriatr Soc. 2005;53(4):695-699. doi:10.1111/j.1532-5415.2005.53221.x 15817019

[26] Blennow Nordström E, Evald L, Mion M, . Combined use of the Montreal Cognitive Assessment and Symbol Digit Modalities Test improves neurocognitive screening accuracy after cardiac arrest: a validation sub-study of the TTM2 trial. Resuscitation. 2024;202:110361. doi:10.1016/j.resuscitation.2024.110361 39147306

[27] Katz MJ, Wang C, Nester CO, . T-MoCA: a valid phone screen for cognitive impairment in diverse community samples. Alzheimers Dement (Amst). 2021;13(1):e12144. doi:10.1002/dad2.12144 33598528 PMC7864219

[28] Pendlebury ST, Welch SJ, Cuthbertson FC, Mariz J, Mehta Z, Rothwell PM. Telephone assessment of cognition after transient ischemic attack and stroke: modified telephone interview of cognitive status and telephone Montreal Cognitive Assessment versus face-to-face Montreal Cognitive Assessment and neuropsychological battery. Stroke. 2013;44(1):227-229. doi:10.1161/STROKEAHA.112.673384 23138443 PMC5593099

[29] Smith A. Symbol Digit Modalities Test. Western Psychological Services; 1982.

[30] Lindvall E, Abzhandadze T, Quinn TJ, Sunnerhagen KS, Lundström E. Is the difference real, is the difference relevant: the minimal detectable and clinically important changes in the Montreal Cognitive Assessment. Cereb Circ Cogn Behav. 2024;6:100222. doi:10.1016/j.cccb.2024.100222 38745691 PMC11090903

[31] Wong GKC, Mak JSY, Wong A, . Minimum clinically important difference of Montreal Cognitive Assessment in aneurysmal subarachnoid hemorrhage patients. J Clin Neurosci. 2017;46:41-44. doi:10.1016/j.jocn.2017.08.039 28887072

[32] Eastwood G, Nichol AD, Hodgson C, ; TAME Study Investigators. Mild hypercapnia or normocapnia after out-of-hospital cardiac arrest. N Engl J Med. 2023;389(1):45-57. doi:10.1056/NEJMoa2214552 37318140

[33] Lachin JM. Worst-rank score analysis with informatively missing observations in clinical trials. Control Clin Trials. 1999;20(5):408-422. doi:10.1016/S0197-2456(99)00022-7 10503801

[34] Rockwood K, Theou O. Using the Clinical Frailty Scale in allocating scarce health care resources. Can Geriatr J. 2020;23(3):210-215. doi:10.5770/cgj.23.463 32904824 PMC7458601

[35] Lövdén M, Fratiglioni L, Glymour MM, Lindenberger U, Tucker-Drob EM. Education and cognitive functioning across the life span. Psychol Sci Public Interest. 2020;21(1):6-41. doi:10.1177/1529100620920576 32772803 PMC7425377

[36] Murman DL. The impact of age on cognition. Semin Hear. 2015;36(3):111-121. doi:10.1055/s-0035-1555115 27516712 PMC4906299

[37] Borland E, Nägga K, Nilsson PM, Minthon L, Nilsson ED, Palmqvist S. The Montreal Cognitive Assessment: normative data from a large Swedish population-based cohort. J Alzheimers Dis. 2017;59(3):893-901. doi:10.3233/JAD-170203 28697562 PMC5545909

[38] Karlsson V, Dankiewicz J, Nielsen N, . Association of gender to outcome after out-of-hospital cardiac arrest—a report from the International Cardiac Arrest Registry. Crit Care. 2015;19(1):182. doi:10.1186/s13054-015-0904-y 25895673 PMC4426639

[39] Agarwal S, Presciutti A, Verma J, . Women have worse cognitive, functional, and psychiatric outcomes at hospital discharge after cardiac arrest. Resuscitation. 2018;125:12-15. doi:10.1016/j.resuscitation.2018.01.036 29407205

[40] Granfeldt A, Holmberg MJ, Nolan JP, Soar J, Andersen LW; International Liaison Committee on Resuscitation ILCOR Advanced Life Support Task Force. Temperature control after adult cardiac arrest: an updated systematic review and meta-analysis. Resuscitation. 2023;191:109928. doi:10.1016/j.resuscitation.2023.109928 37558083

[41] Kiguchi T, Okubo M, Nishiyama C, . Out-of-hospital cardiac arrest across the world: first report from the International Liaison Committee on Resuscitation (ILCOR). Resuscitation. 2020;152:39-49. doi:10.1016/j.resuscitation.2020.02.044 32272235

[42] Caro-Codón J, Rey JR, Lopez-de-Sa E, . Long-term neurological outcomes in out-of-hospital cardiac arrest patients treated with targeted-temperature management. Resuscitation. 2018;133:33-39. doi:10.1016/j.resuscitation.2018.09.015 30253227

[43] Jaeschke R, Singer J, Guyatt GH. Measurement of health status: ascertaining the minimal clinically important difference. Control Clin Trials. 1989;10(4):407-415. doi:10.1016/0197-2456(89)90005-6 2691207

[44] Copay AG, Subach BR, Glassman SD, Polly DW Jr, Schuler TC. Understanding the minimum clinically important difference: a review of concepts and methods. Spine J. 2007;7(5):541-546. doi:10.1016/j.spinee.2007.01.008 17448732

[45] Baldi E, Wnent J, Caputo ML, . European Resuscitation Council guidelines 2025: epidemiology in resuscitation. Resuscitation. 2025;215(suppl 1):110733. doi:10.1016/j.resuscitation.2025.110733 41117565

[46] Haywood K, Whitehead L, Nadkarni VM, ; COSCA Collaborators. COSCA (Core Outcome Set for Cardiac Arrest) in adults: an advisory statement from the International Liaison Committee on Resuscitation. Circulation. 2018;137(22):e783-e801. doi:10.1161/CIR.0000000000000562 29700122

[47] Martin SS, Aday AW, Allen NB, ; American Heart Association Council on Epidemiology and Prevention Statistics Committee and Stroke Statistics Committee. 2025 heart disease and stroke statistics: a report of US and global data from the American Heart Association. Circulation. 2025;151(8):e41-e660. doi:10.1161/CIR.0000000000001303 39866113 PMC12256702

