# Supplementary material for: Long-Term Outcomes and Recovery Trajectories in Out-of-Hospital Cardiac Arrest: A 2-Year Follow-Up of the Randomized Clinical TTM2 Trial
Source: JAMA Neurol. 2026 Feb 16;83(4):339–47. doi: 10.1001/jamaneurol.2025.5614 (PMC12910457; doi:10.1001/jamaneurol.2025.5614)
Supplement: Supplement 1. — eMethods 1. Sites in the TTM2 trial eMethods 2. Changes From the Published Protocol eMethods 3. R-packages Used for Statistical analyses and Figures eTable 1. Analysis Comparing Functional Outcome and Cognitive Function in Out-of-Hospital Cardiac Arrest Survivors Treated with Hypothermia versus Normothermia eTable 2. Functional and Cognitive Outcomes at 6 and 24 Months, stratified by temperature group eTable 3. Participant Characteristics Stratified by Change in Assessment Scores Between 6 & 24 Months eTable 4. Occupational status, Pre-arrest Workers eTable 5. Item-specific Results on the MoCA-30 eTable 6. Analysis Exploring Impact of Age, Education and Sex on Cognitive Outcome eFigure. CONSORT Flowchart of Participants and Loss to-follow-up eReferences [file jamaneurol-e255614-s001.pdf]

Supplemental Online Content

Hultgren M, Nordström EB, Ullén S, et al. Long-term outcomes and recovery trajectories in out-of-hospital cardiac arrest: a 2-year follow-up of the randomized clinical TTM2 trial. *JAMA Neurol.* Published online February 16, 2026.
doi:10.1001/jamaneurol.2025.5614

eMethods 1. Sites in the TTM2 trial
eMethods 2. Changes From the Published Protocol
eMethods 3. R-packages Used for Statistical analyses and Figures
eTable 1. Analysis Comparing Functional Outcome and Cognitive Function in Out-of-Hospital Cardiac Arrest Survivors Treated with Hypothermia versus Normothermia
eTable 2. Functional and Cognitive Outcomes at 6 and 24 Months, stratified by temperature group
eTable 3. Participant Characteristics Stratified by Change in Assessment Scores Between 6 & 24 Months
eTable 4. Occupational status, Pre-arrest Workers
eTable 5. Item-specific Results on the MoCA-30
eTable 6. Analysis Exploring Impact of Age, Education and Sex on Cognitive Outcome
eFigure. CONSORT Flowchart of Participants and Loss to-follow-up
eReferences

This supplemental material has been provided by the authors to give readers additional information about their work.

© 2026 American Medical Association. All rights reserved, including those for text and data mining, AI training, and similar technologies.

eMethods 1. Sites in the TTM2 trial

Australia: Royal North Shore Hospital, Sydney; Concord Repatriation General Hospital, Sydney; The Alfred Hospital, Melbourne; Nepean Hospital, Sydney; Northern Hospital, Melbourne; Austin Hospital, Melbourne; St. Vincent's Hospital, Sydney; Princess Alexandra Hospital, Brisbane; John Hunter Hospital, Newcastle; Liverpool Hospital, Sydney.
Austria: Medical University Innsbruck, Innsbruck.
Belgium: Erasme University Hospital, Brussels; Ziekenhuis Oost-Limburg, Genk.
Czech Republic: General University Hospital, Prague; University Hospital Hradec Králové, Hradec Králové; Regional Hospital Liberec, Liberec.
Denmark: Aarhus University Hospital, Aarhus.
France: Cochin University Hospital (APHP), Paris; Lariboisiere University Hospital (APHP), Paris; Centre Hospitalier de Versailles, Le Chesnay; CHU de Nantes, Nantes; Dupuytren Teaching Hospital, Limoges.
Germany: Charitè University Hospital, Berlin.
Italy: San Martino Policlinico Hospital, Genoa; Civil Hospital, Baggiovara, Modena; Trieste University Hospital, Trieste.
New Zealand: Wellington Regional Hospital, Wellington; Christchurch Hospital, Christchurch.
Norway: Oslo University Hospital, Rikshospitalet, Oslo; St Olav's University Hospital, Trondheim; Sorlandet Hospital, Arendal; Haukeland University Hospital, Bergen.
Sweden: Skåne University Hospital, Malmö; Skåne University Hospital, Lund; Helsingborg Hospital, Helsingborg; Södersjukhuset, Karolinska Institutet, Stockholm; Capio Sankt Göran Hospital, Stockholm; Sahlgrenska University Hospital, Göteborg; Uppsala University Hospital, Uppsala; Linköping University Hospital, Linköping; Northern Älvsborg County Hospital, Trollhättan; Skaraborg Hospital, Skövde; Örebro University Hospital, Örebro; Halland hospital, Halmstad; Karlstad Central Hospital, Karlstad.
Switzerland: Lausanne University Hospital, Lausanne; Bern University Hospital, Bern; Cardiocentro Ticino, Lugano; Cantonal Hospital St. Gallen, St Gallen; University Hospital Zurich, Zurich.
United Kingdom: University Hospital of Wales, Cardiff; Royal Victoria Hospital, Belfast; Essex Cardiothoracic Centre MSENHSFT, Basildon; Royal Berkshire Hospital, Reading; Manchester Royal Infirmary, Manchester; Bristol Royal Infirmary, Bristol; University Hospital Birmingham NHS foundation Trust, Birmingham; Queen Alexandra Hospital, Portsmouth; Royal Bournemouth Hospital, Bournemouth.
USA: University of Pittsburgh, Pittsburgh; Mayo Clinic, Rochester.

© 2026 American Medical Association. All rights reserved, including those for text and data mining, AI training, and similar technologies.

eMethods 2. Changes From the Published Protocol

The statistical methods for temperature group comparisons were performed using the same methods at 6 and 24 months. Changes compared to the published protocol were as follows:

- Change: Age, education and version adjusted z-scores were used for the SDMT instead of raw scores (0-110). Reason: Both the oral and written version was used during follow-up and have different normative values. To enable combined analyses of the SDMT versions, z-scores were used instead. Consequence: Temperature group comparisons with SDMT were not adjusted for age and education as it was already accounted for in the test scores.
- Change: The stratified Wilcoxon Mann Whitney U test was used instead of the prespecified non-parametric van Elteren test for initial temperature group comparisons (including deceased) for MoCA and SDMT. Reason: The non-parametric van Elteren test was unavailable in the program *R: language and environment for statistical computing*.
- Change: Not all descriptive information was presented stratified by intervention groups. Reason: Analyses on recovery trajectories stratified by temperature groups were planned if significant differences were found between the intervention groups in the temperature group comparisons. Due to the longitudinal design with multiple points of follow-up, it was deemed more appropriate to only present results stratified by intervention groups where it was most relevant to facilitate interpretation of the results.
- Change: The specific statistical analyses (including descriptive statistics and linear regression analyses) that were used to explore recovery trajectories were not pre-specified. Reason: It was pre-specified to explore the course of recovery, and these analyses were included to provide detailed data on recovery trajectories.

© 2026 American Medical Association. All rights reserved, including those for text and data mining, AI training, and similar technologies.

eMethods 3. R-packages Used for Statistical analyses and Figures

- ggplot2[1]
- ggthemes[2]
- haven[3]
- nlme[4]
- ordinal[5]
- paletteer[6]
- patchwork[7]
- readr[8]
- RColorBrewer[9]
- sanon[10]
- tidyverse[11]

© 2026 American Medical Association. All rights reserved, including those for text and data mining, AI training, and similar technologies.

eTable 1. Analysis Comparing Functional Outcome and Cognitive Function in Out-of-Hospital Cardiac Arrest Survivors Treated with Hypothermia versus Normothermia

Outcome assessment	[a,b]All, including deceased. Estimate (CI)	[a]All, including deceased. *P*-value.	[c]Model 1: Odds ratio/Mean difference (CI)	Model 1: *P*-value	[d]Model 2: Odds ratio/Mean difference (CI)	Model 2: *P*-value
[e]GOSE	-0.01 (-0.03 to 0.02)	0.68	0.98 (0.73 to 1.31)	0.88	0.97 (0.72 to 1.30)	0.84
[f]MoCA-30	-0.02 (-0.04 to 0.01)	0.27	0.12 (-0.54 to 0.79)	0.72	-0.02 (-0.67 to 0.63)	0.95
[g]SDMT	-0.01 (-0.03 to 0.02)	0.65	-0.06 (-0.31 to 0.18)	0.62	-0.09 (-0.33 to 0.16)	0.49

The reference group for the analyses was normothermia.

[a]Stratified Wilcoxon Mann Whitney U test. Adjusted for site and co-enrollment in the TAME trial. No. for each outcome n=1638, n=1577, n=1358 for GOSE, MoCA and SDMT respectively.

[b]The estimates are the Mann-Whitney estimator subtracted with 0.5.

[c]Mixed effects ordinal regression used for GOSE, and mixed effects linear regression used for MoCA and SDMT. Model 1 included adjustment for site and co-enrollment in the TAME trial. No. for each outcome n=670, n=609, n=390 for GOSE, MoCA and SDMT respectively.

[d]Mixed effects ordinal regression used for GOSE, and mixed effects linear regression used for MoCA and SDMT. Model 2 included adjustment for site, co-enrollment in the TAME trial, age (younger/older than 65 years of age), education (any university studies yes/no), sex (male/female), and pre-arrest Clinical Frailty Scale score (Not frail:1-4; Frail: 5-9). No. for each outcome n=670, n=609, n=390 for GOSE, MoCA and SDMT respectively.

[e]Reported as odds ratio.

[f]In Model 2 education was excluded. Includes converted telephone MoCA.

[g]In Model 2 age and education was excluded.

Abbreviations: GOSE, Glasgow Outcome Scale Extended; MoCA, Montreal Cognitive Assessment; Symbol Digit Modalities Test; CI, confidence interval.

© 2026 American Medical Association. All rights reserved, including those for text and data mining, AI training, and similar technologies.

eTable 2. Functional and Cognitive Outcomes at 6 and 24 Months, stratified by temperature group

**Outcome**	**6-month follow-up**		**24-month follow-up**	
	Hypothermia	Normothermia	Hypothermia	Normothermia
GOSE No.	*415*	*419*	*332*	*338*
GOSE≤6	178 (43)	168 (40)	99 (30)	92 (27)
[a]MoCA-30 No.	*384*	*376*	*297*	*312*
[a]MoCA-30, median (IQR)	27 (23-29)	26 (23-28)	26 (23-28)	26 (23-28)
[a]MoCA-30 <26, No. (%)	158 (41)	172 (46)	139 (46)	139 (45)
MoCA No.	*305*	*302*	*191*	*207*
MoCA, median (IQR)	27 (24-29)	26 (23-28)	27 (23-29)	26 (23-28)
MoCA <26, No. (%)	117 (38)	131 (43)	71 (37)	86 (42)
T-MoCA No.	*79*	*74*	*106*	*105*
T-MoCA median (IQR)	19 (17-21)	19 (17-21)	19 (16-20)	20 (17-21)
T-MoCA <19, No. (%)	32 (41)	29 (39)	48 (45)	39 (37)
SDMT No.	*303*	*298*	*188*	*202*
SDMT z-score, median (IQR)	-0.91 (-1.78 to -0.11)	-0.96 (-1.97 to -0.18)	-0.77 (-1.5 to -0.10)	-0.69 (-1.7 to -0.11)
SDMT z-score, mean (SD)	-1.01 (1.39)	-1.09 (1.36)	-0.84 (1.3)	-0.9 (1.3)
SDMT ≤−1 SD, No. (%)	142 (47)	144 (48)	81 (43)	83 (41)
SDMT ≤−1.5 SD, No. (%)	90 (28)	103 (35)	49 (26)	58 (29)
MoCA-30 and SDMT No.	*302*	*298*	*188*	*202*
[a]MoCA-30 **OR** SDMT under cut-off, No. (%)	176 (58)	177 (60)	106 (56)	115 (57)
[a]MoCA-30 **AND** SDMT under cut-off, No. (%)	82 (27)	94 (32)	44 (23)	53 (26)

[a]Includes converted T-MoCA.
Continuous data are presented as median with IQR or as mean with SD. Categorical and binary data are presented as frequency (No.) with percentages (%). Abbreviations: GOSE, Glasgow Outcome Scale Extended; No, number; MoCA, Montreal Cognitive Assessment; IQR, interquartile range; T-MoCA, Telephone MoCA; SDMT, Symbol Digit Modalities Test; SD, standard deviation.

© 2026 American Medical Association. All rights reserved, including those for text and data mining, AI training, and similar technologies.

eTable 3. Participant Characteristics Stratified by Change in Assessment Scores Between 6 & 24 Months

Variable	Decline			No Change			Improvement		
	[a]*GOSE*	[b]*MoCA-30*	*SDMT*	[a]*GOSE*	[b]*MoCA-30*	*SDMT*	[a]*GOSE*	[b]*MoCA-30*	*SDMT*
**General pre-OHCA, No. (%)**									
Age, years (SD)	65 (12)	60 (13)	60 (12)	59 (14)	60 (13)	61 (14)	58 (13)	58 (15)	57 (14)
Male sex	121 (84)	164 (85)	104 (87)	287 (85)	224 (85)	68 (84)	178 (84)	92 (81)	124 (80)
University-level education	38 (27)	48 (25)	38 (32)	112 (34)	103 (39)	32 (40)	77 (36)	48 (42)	50 (32)
**Medical history (prior to cardiac arrest), No. (%)**									
Charlson Comorbidity Index, median (IQR)	3 (2-4)	2 (1-3)	2 (1-3)	2 (1-3)	2 (1-3)	2 (1-3)	2 (1-3)	2 (1-3)	2 (1-3)
Pre-arrest Frailty (CFS >4)	5 (3)	2 (1)	2 (2)	4 (1)	4 (2)	3 (4)	1 (<1)	1 (<1)	1 (<1)
Diabetes	29 (20)	28 (14)	14 (12)	38 (11)	25 (10)	11 (14)	31 (15)	12 (10)	19 (12)
Heart failure	12 (8)	13 (7)	11 (9)	16 (5)	15 (6)	4 (5)	12 (6)	3 (3)	8 (5)
Hypertension with pharmacological treatment	47 (33)	56 (30)	43 (37)	106 (33)	88 (35)	29 (38)	64 (31)	31 (30)	46 (31)
Known neurological disease	17 (12)	10 (5)	6 (5)	23 (7)	20 (8)	8 (10)	12 (6)	1 (9)	10 (6)
Memory problems (self-reported)	12 (8)	13 (7)	8 (7)	31 (9)	22 (8)	9 (11)	12 (6)	8 (7)	10 (6)
Myocardial infarction	32 (22)	25 (13)	15 (13)	38 (12)	35 (14)	15 (19)	33 (16)	14 (14)	21 (14)
**OHCA resuscitation variables, No. (%)**									
Location of cardiac arrest, at home	63 (44)	79 (41)	49 (41)	144 (43)	117 (44)	36 (44)	93 (44)	46 (40)	70 (45)
Bystander-witnessed arrest	134 (93)	180 (93)	114 (95)	313 (93)	245 (93)	78 (96)	197 (92)	105 (92)	137 (88)
First monitored rhythm, shockable	122 (85)	173 (89)	110 (92)	306 (91)	240 (91)	74 (91)	196 (92)	105 (92)	138 (89)
Time (minutes) to sustained ROSC, median (IQR)	22 (14-38)	20 (14-30)	19 (13-29)	20 (14-29)	20 (14-30)	21 (16-32)	22 (15-30)	20 (13-30)	22 (15-30)
**Data on hospital admission, No. (%)**									
Shock	28 (19)	33 (17)	27 (23)	64 (19)	52 (20)	21 (26)	46 (22)	24 (21)	33 (21)
**In-hospital**									
Days in hospital, median (IQR)	17 (11-31)	16 (11-27)	16 (11-27)	15 (10-22)	13 (10-19)	14 (9-19)	16 (11-25)	17 (11-26)	14 (9-22)
Days in intensive care unit, median (IQR)	5 (4-10)	6 (4-10)	5 (3-10)	5 (3-8)	5 (3-8)	5 (3-8)	6 (3-10)	5 (3-8)	4 (3-8)
**Rehabilitation provided (self-reported) at 6-month follow-up, No. (%)**									
[c]Cardiac rehabilitation	31 (22)	52 (27)	38 (32)	92 (27)	84 (32)	19 (23)	72 (34)	29 (25)	50 (32)
[c]Exercise-based cardiac rehabilitation	31 (22)	53 (27)	22 (18)	69 (20)	56 (21)	19 (24)	47 (22)	19 (17)	26 (17)

© 2026 American Medical Association. All rights reserved, including those for text and data mining, AI training, and similar technologies.

eTable 3. Participant Characteristics Stratified by Change in Assessment Scores Between 6 & 24 Months (continued)

Variable	Decline			No Change			Improvement		
	[a]*GOSE*	[b]*MoCA-30*	*SDMT*	[a]*GOSE*	[b]*MoCA-30*	*SDMT*	[a]*GOSE*	[b]*MoCA-30*	*SDMT*
[c]Inpatient neurological/cognitive/brain injury rehabilitation	23 (16)	20 (10)	19 (16)	35 (10)	24 (9)	11 (14)	25 (12)	17 (15)	21 (14)
[c]Outpatient neurological/cognitive/brain injury rehabilitation	12 (8)	12 (6)	12 (10)	15 (4)	20 (8)	7 (9)	16 (8)	4 (4)	10 (6)
[c]Other	10 (7)	17 (9)	5 (4)	16 (5)	6 (2)	4 (5)	12 (6)	7 (6)	8 (5)

[a]Includes participants alive at 6 months

[b]Includes converted telephone MoCA

[c]Categories are not mutually exclusive. Participants may have participated in multiple different categories of rehabilitation.

Table comparing age, education and sex for GOSE, MoCA-30, and SDMT between 6 and 24 months stratified by the minimal important difference (1 category for GOSE, 2 points for MoCA-30 & 0.2 z-score for SDMT). Continuous data are presented as median with IQR or as mean with SD. Categorical and binary data are presented as frequency (No.) with percentages (%).

Abbreviations: GOSE, Glasgow Outcome Scale Extended; MoCA, Montreal Cognitive Assessment; SDMT, Symbol Digit Modalities Test; No, number; SD, standard deviation; IQR, interquartile range; CFS, Clinical Frailty Scale; OHCA, out-of-hospital cardiac arrest; ROSC, return of spontaneous circulation.

© 2026 American Medical Association. All rights reserved, including those for text and data mining, AI training, and similar technologies.

eTable 4. Occupational status, Pre-arrest Workers

Occupational status, No. (%)	Pre-arrest		6-month follow-up		24-month follow-up	
	Hypothermia	Normothermia	Hypothermia	Normothermia	Hypothermia	Normothermia
No.	231	207	231	207	177	175
[a]Full-time	192 (83)	175 (85)	87 (38)	95 (46)	92 (52)	105 (60)
[b]Part-time	39 (17)	32 (16)	53 (23)	40 (19)	23 (13)	29 (17)
Unemployed	N/A	N/A	13 (6)	6 (3)	11 (6)	4 (2)
Retired (age)	N/A	N/A	9 (4)	3 (1)	20 (11)	13 (7)
Retired (sick/dis.)	N/A	N/A	19 (8)	8 (4)	20 (11)	19 (11)
Sick leave	N/A	N/A	47 (20)	48 (23)	6 (3)	5 (6)
Other (e.g. student)	N/A	N/A	3 (1)	7 (3)	5 (3)	0 (0)
Return to work, days, median (IQR)	N/A	N/A	80 (50-110)	76 (46-118)	104 (62-212)	106 (61-215)
Return to work, days, mean (SD)	N/A	N/A	85 (47)	87 (53)	169 (161)	175 (168)

[a]Full-time defined as 30h or more/week.
[b]Part-time defined as less than 30h/week.
Continuous data are presented as median with IQR or as mean with SD. Categorical and binary data are presented as frequency (No.) with percentages (%). Abbreviations: No, number; N/A, not applicable; IQR, interquartile range; SD, standard deviation.

© 2026 American Medical Association. All rights reserved, including those for text and data mining, AI training, and similar technologies.

eTable 5. Item-specific Results on the MoCA-30

MoCA-30 Item	Item score range	6-month mean (SD)	Percent of maximum score, %	24-month mean (SD)	Percent of maximum score, %	[a]Mean change in item score
[b]Visuospatial/Executive	0-5	4.01 (1.26)	80	4.2 (1.42)	84	0.19
[b]Naming	0-3	2.88 (0.46)	96	2.92 (0.34)	97	0.04
Attention – Digits	0-2	1.75 (0.51)	88	1.78 (0.47)	89	0.03
Attention - Letters	0-1	0.93 (0.26)	93	0.96 (0.20)	96	0.03
Attention - Subtraction	0-3	2.66 (0.77)	89	2.68 (0.73)	89	0.02
Language - Repetition	0-2	1.62 (0.66)	81	1.62 (0.64)	81	0.00
Language - Fluency	0-1	0.59 (0.49)	59	0.61 (0.49)	61	0.02
Abstraction	0-2	1.64 (0.62)	82	1.72 (0.55)	86	0.08
Memory - Delayed recall	0-5	2.95 (1.72)	59	3.23 (1.62)	65	0.28
Orientation	0-6	5.67 (0.97)	95	5.75 (0.73)	96	0.08

[a]24-month mean compared to 6-month mean.
[b]Not included in T-MoCA. Mean score based on face-to-face MoCA only.
Abbreviations: MoCA, Montreal Cognitive Assessment; SD, standard deviation.

© 2026 American Medical Association. All rights reserved, including those for text and data mining, AI training, and similar technologies.

eTable 6. Analysis Exploring Impact of Age, Education and Sex on Cognitive Outcome

[a]Explanatory variable	[b]MoCA-30, mean difference (95% CI) n=571	R^2	*P*-value	[c]SDMT, mean difference (95% CI) n=356	R^2	*P*-value
Age	-0.24 (-0.75 to 0.27)	0.03	<.001	N/A	0.01	.02
Education	1.01 (0.51 to 1.52)*			N/A		
Sex	0.05 (-0.60 to 0.70)			-0.30 (-0.55 to -0.06)*		

Linear regression was used.

[a]Younger/older than 65 years of age, education (any university studies yes/no), sex (male/female). Reference categories <65 years, no university education and male sex.
[b]Includes converted telephone MoCA.
[c]Age and education already accounted for in scoring.
*Indicates significant variable. A *P*-value <.05 was considered significant.
Abbreviations: MoCA, Montreal Cognitive Assessment; Symbol Digit Modalities Test; CI, confidence interval; N/A, not applicable.

© 2026 American Medical Association. All rights reserved, including those for text and data mining, AI training, and similar technologies.

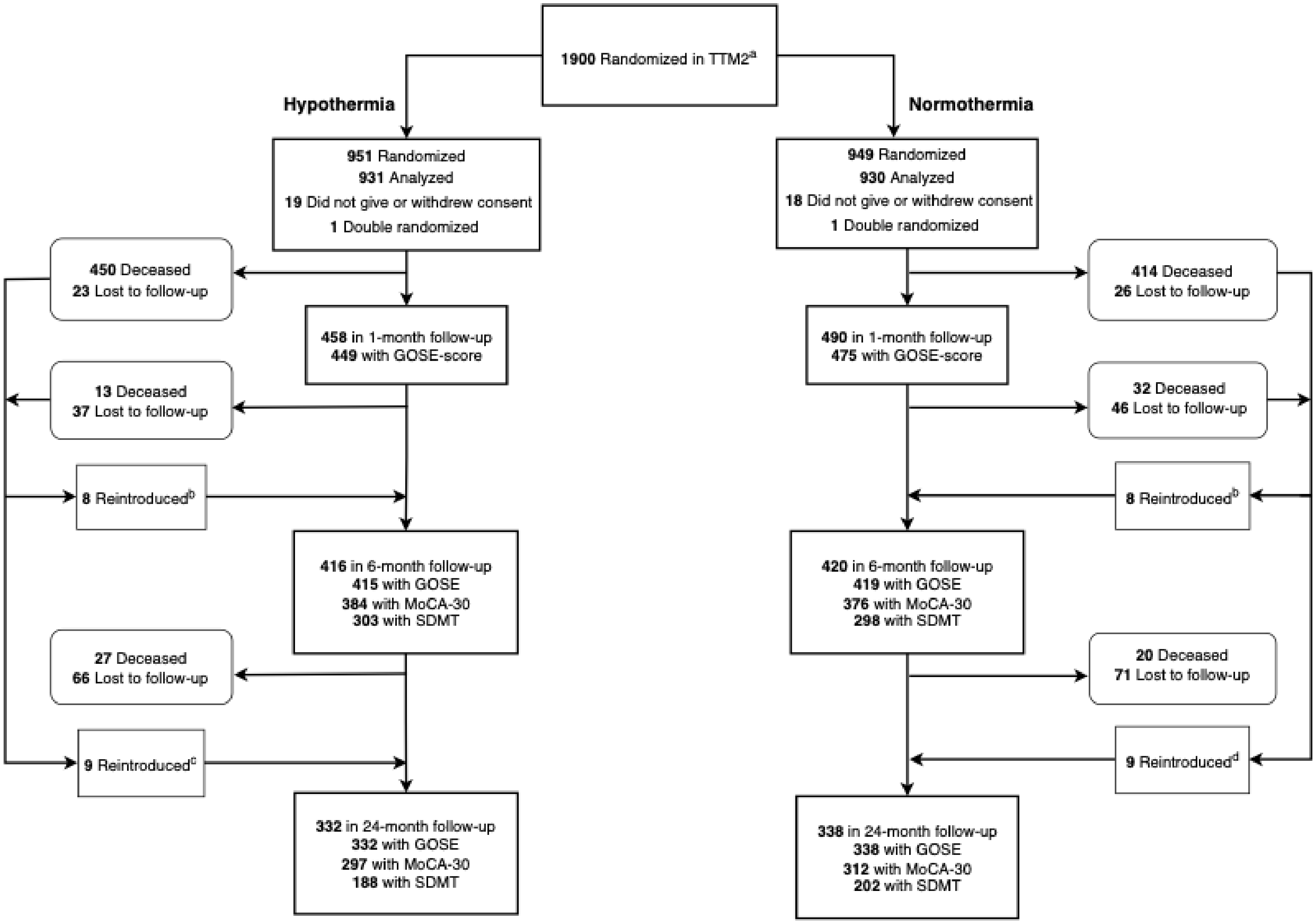

© 2026 American Medical Association. All rights reserved, including those for text and data mining, AI training, and similar technologies.

eFigure. CONSORT Flowchart of Participants and Loss to-follow-up

[a]For participants screened for eligibility and reasons for exclusion, please see the main TTM2 trial or 6-month follow-up.[12,13]
[b]Reintroduced into follow-up at 6 months, not in 1-month follow-up.
[c]Reintroduced into follow-up at 24 months. Seven participated at 1 month but not 6 months, two participated only at 24 months.
[d]Reintroduced into follow-up at 24 months. Nine participated at 1 month but not 6 months.
Abbreviations: TTM2, Targeted Hypothermia vs Targeted Normothermia After Out-of-Hospital Cardiac Arrest trial; GOSE, Glasgow Outcome Scale Extended; MoCA, Montreal Cognitive Assessment; SDMT, Symbol Digit Modalities Test.

© 2026 American Medical Association. All rights reserved, including those for text and data mining, AI training, and similar technologies.

eReferences:

1. H. Wickham. ggplot2: Elegant Graphics for Data Analysis. Springer-Verlag New York, 2016.

2. Arnold J (2024). _ggthemes: Extra Themes, Scales and Geoms for 'ggplot2'_. doi:10.32614/CRAN.package.ggthemes https://doi.org/10.32614/CRAN.package.ggthemes, R package version 5.1.0, https://CRAN.R-project.org/package=ggthemes.

3. Wickham H, Miller E, Smith D (2025). _haven: Import and Export 'SPSS', 'Stata' and 'SAS' Files_. doi:10.32614/CRAN.package.haven https://doi.org/10.32614/CRAN.package.haven, R package version 2.5.5, https://CRAN.R-project.org/package=haven.

4. Pinheiro J, Bates D, R Core Team (2025). _nlme: Linear and Nonlinear Mixed Effects Models_. https://doi.org/10.32614/CRAN.package.nlme, R package version 3.1-168, https://CRAN.R-project.org/package=nlme.

5. Christensen R (2023). *ordinal—Regression Models for Ordinal Data*. R package version 2023.12-4.1, https://CRAN.R-project.org/package=ordinal.

6. Hvitfeldt E. (2021). paletteer: Comprehensive Collection of Color Palettes. version 1.3.0. https://github.com/EmilHvitfeldt/paletteer.

7. Pedersen T (2025). _patchwork: The Composer of Plots_. doi:10.32614/CRAN.package.patchwork https://doi.org/10.32614/CRAN.package.patchwork, R package version 1.3.2, <https://CRAN.R-project.org/package=patchwork>.

8. Wickham H, Hester J, Bryan J (2024). _readr: Read Rectangular Text Data_. doi:10.32614/CRAN.package.readr https://doi.org/10.32614/CRAN.package.readr, R package version 2.1.5, https://CRAN.R-project.org/package=readr.

9. RColorBrewer. Neuwirth E (2022). _RColorBrewer: ColorBrewer Palettes_. doi:10.32614/CRAN.package.RColorBrewer https://doi.org/10.32614/CRAN.package.RColorBrewer, R package version 1.1-3, https://CRAN.R-project.org/package=RColorBrewer.

10. Kawaguchi A, Koch GG (2015). "sanon: An R Package for Stratified Analysis with Nonparametric Covariable Adjustment." *Journal of Statistical Software*, **67**(9), 1–37. doi:10.18637/jss.v067.i09.

11. Wickham H, Averick M, Bryan J et al (2019). "Welcome to the tidyverse." _Journal of Open Source Software_, *4*(43), 1686. doi:10.21105/joss.01686 https://doi.org/10.21105/joss.01686.

12. Dankiewicz J, Cronberg T, Lilja G, et al. Hypothermia versus Normothermia after Out-of-Hospital Cardiac Arrest. *New England Journal of Medicine*. 2021;384(24):2283–2294. doi:10.1056/NEJMoa2100591

13. Lilja G, Ullén S, Dankiewicz J, et al. Effects of Hypothermia vs Normothermia on Societal Participation and Cognitive Function at 6 Months in Survivors After Out-of-Hospital Cardiac Arrest: A Predefined Analysis of the TTM2 Randomized Clinical Trial. *JAMA Neurology*. 2023;80(10):1070–1079. doi:10.1001/jamaneurol.2023.2536

© 2026 American Medical Association. All rights reserved, including those for text and data mining, AI training, and similar technologies.
