# Supplementary material for: Long-Term Outcomes and Recovery Trajectories in Out-of-Hospital Cardiac Arrest: A 2-Year Follow-Up of the Randomized Clinical TTM2 Trial
Source: JAMA Neurol. 2026 Feb 16;83(4):339–47. doi: 10.1001/jamaneurol.2025.5614 (PMC12910457; doi:10.1001/jamaneurol.2025.5614)
Supplement: Supplement 2. — Trial Protocol [file jamaneurol-e255614-s002.pdf]

ttm2

Hypothermia or Early Treatment of Fever

Targeted Hypothermia versus Targeted Normothermia after Out-of-hospital Cardiac Arrest. A Randomised Clinical Trial

Version 1.1
November 21, 2017

Short title:
Targeted Temperature Management after Cardiac Arrest 2

Clinical trials identifier:
NCT02908308

Sponsor:
Region Skåne - Skånevård SUND - Helsingborgs Hospital
S Vallgatan 5, 251 87 Helsingborg, Sweden
Chief Investigator: Niklas Nielsen, MD, PhD, DEAA, EDIC
Department of Anaesthesiology and Intensive Care Helsingborg Hospital
Lund University, Faculty of Medicine

PROTOCOL TITLE:
Targeted Hypothermia versus Targeted Normothermia after Out-of-hospital Cardiac Arrest.
A Randomised Clinical Trial

Chief Investigator and Study Chair:
Niklas Nielsen, MD, PhD
Region Skåne, Lund Unviersity

Protocol Version: 1.1
Version Date: November 21, 2017

I confirm that I have read this protocol and that I understand it. I will conduct the study according to the protocol and according to the ethical principels that have their origin in the World Medical Association's Declaration of Helsinki.

Principal Investigator Name: Niklas Nielsen

Principal Investigator Signature:

Date: Nov-21-2017

Contents

List of terms 5

1 Trial Overview 6

2 Background and Significance 7
2.1 Randomised Trials . . . 7
2.2 Hypothermia in other areas . . . 8
2.3 Rationale for a new trial . . . 8
2.4 Rationale for early treatment of fever . . . 9

3 Trial hypotheses and outcomes 11
3.1 Primary outcome . . . 11
3.2 Secondary outcomes . . . 11
3.3 Explorative outcomes . . . 11
3.4 Rationale for chosen outcomes . . . 11

4 Eligibility 13
4.1 Inclusion criteria . . . 13
4.2 Exclusion criteria . . . 13
4.3 Note on inclusion and exclusion criteria . . . 13
4.4 Note on inclusion window . . . 14
4.5 Exit from the trial . . . 15

5 Trial design 16
5.1 Screening and randomisation (Phase 1) . . . 16
5.2 Intervention period (Phase 2) . . . 16
5.3 After the intervention period (Phase 3) . . . 16
5.4 General ICU-care . . . 17
5.4.1 Sedation . . . 17
5.4.2 Shivering . . . 17
5.4.3 The Bedside Shivering Assessment Scale (BSAS) . . . 18
5.5 Prognostication . . . 18
5.5.1 Clinical examination - mandatory . . . 18
5.5.2 EEG - mandatory . . . 19
5.5.3 Brain CT - optional . . . 19
5.5.4 Brain MRI - optional . . . 19
5.5.5 Neuron specific enolase- optional . . . 19
5.5.6 SSEP - optional . . . 19
5.5.7 The TTM2-trial criteria for a likely poor neurological outcome . . . 20
5.6 Withdrawal of life supporting therapies (WLST) . . . 21
5.6.1 Brain death . . . 21
5.7 Follow up . . . 21
5.8 Blinding . . . 22

6 Coenrolment 23
6.1 Coenrolment in the TAME trial . . . 23
6.1.1 Stratification . . . 24
6.2 Coenrolment in other trials . . . 24

7 Intervention period (Phase 2) 25
7.1 Hypothermia . . . 25
7.1.1 Block A - cooling phase and maintenance phase . . . 25
7.1.2 Block B - Rewarming . . . 28
7.1.3 Early termination of the intervention . . . 28
7.1.4 After rewarming . . . 28
7.2 Normothermia and early treatment of fever . . . 28

8 Data collection 30
8.1 Baseline data . . . 30
8.1.1 Pre-randomisation characteristics . . . 30
8.1.2 Pre-hospital data . . . 30
8.1.3 Background data . . . 31
8.2 Data on hospital admission . . . 31
8.3 In the ICU . . . 32
8.3.1 Data during the intervention . . . 32
8.3.2 Additional temperature measurements . . . 32
8.3.3 Daily during the ICU stay: . . . 32
8.3.4 Neurology . . . 32
8.3.5 At ICU discharge . . . 33
8.4 At hospital discharge . . . 33
8.5 30 days after randomisation . . . 33
8.6 180 days after randomisation . . . 33
8.7 24 months after randomisation . . . 33
8.8 Planned investigations . . . 33
8.9 Laboratory testing . . . 34
8.10 Biobank . . . 34
8.10.1 Blood samples . . . 34

9 Ethics and informed consent 35

10 Data management 36
10.1 Data handling and record keeping . . . 36
10.2 Quality control and quality assurance . . . 36

11 Adverse events 37
11.1 Definitions . . . 37
11.2 Reporting of serious adverse events . . . 38

12 Statistical plan and data analysis 40
12.1 Sample size . . . 40
12.2 Analysis methods . . . 40
12.2.1 Primary outcome . . . 40
12.2.2 Secondary outcome . . . 40
12.3 Missing data . . . 40
12.4 Subgroup analysis . . . 41
12.5 Data safety monitoring committee . . . 41
12.5.1 Members of the DSMC-committee . . . 41

13 Publication of Data 42

14 Insurance **42**

15 Funding **42**

16 Timeline **43**

17 Trial Participants **44**
17.1 Steering Group . . . 44
17.2 Investigators - TBD . . . 45
17.3 Investigator responsibilities . . . 46

Appendix A The FOUR SCORE **52**

Appendix B The modified Rankin scale (mRS) **53**

Appendix C The Glasgow outcome scale - extended (GOSE) **53**

Appendix D Charter for the DSMC of the TTM2-trial **54**
D.1 Introduction . . . 54
D.2 Primary responsibilities of the DSMC . . . 54
D.3 Members of the DSMC . . . 54
D.3.1 Members of the DSMC-committee . . . 55
D.4 Conflicts of interest . . . 55
D.5 Formal interim analysis meeting . . . 55
D.6 Proper communication . . . 55
D.7 Closed Sessions . . . 55
D.8 Open Reports . . . 56
D.9 Minutes of the DSMC Meetings . . . 56
D.10 Recommendations to the Steering Committee . . . 56
D.11 Statistical monitoring guidelines . . . 56
D.12 Conditions for transfer of data from the Coordinating Centre to the DSMC . . . 57

Appendix E Version History **58**

Acronyms

AE	Adverse Event
AHA	American Heart Association
BSAS	Bedside shivering assessment scale
CA	Cardiac arrest
CPC	Cerebral Performance Category
DNR	Do Not Resuscitate
DSMC	Data Safety Monitoring Committee
eCRF	electronic Case Report Form
EQ5D-5L	Euroqol health Survey 5 Dimensions 5 Level version
ERC	European Resuscitation Council
GOS	Glasgow Outcome Scale
GOS-E	Glasgow Outcome Scale-Extended
HRQoL	Health-Related Quality of Life
IHCA	In-hospital cardiac arrest
ILCOR	International Liaison Committee on Resuscitation
IQCODE	Informant Questionnaire on Cognitive Decline in the Elderly
MoCA	Montreal Cognitive Assessment
mRS	modified Rankin Scale
NSE	Neuronspecific enolase
OHCA	out-of-hospital cardiac arrest
RASS	Richmond Agitation-Sedation Scale
ROSC	return of spontaneous circulation
SAE	Serious Adverse Event
SBU	Statens beredning för medicinsk och social utvärdering - Swedish agency for health technology assessment and assessment of social services
SDMT	Symbol Digit Modalities Test
TSQ	Two Simple Questions
WLST	Withdrawal of Life Supporting Therapies

1 Trial Overview

The TTM2 trial is a continuation of the collaboration that resulted in the previous Target Temperature Management after out-of-hospital cardiac arrest trial (hereafter: TTM1). With its planned size TTM2 will supersede the TTM1 trial as the largest trial on temperature management as a post-cardiac arrest intervention.

The TTM1 trial (NCT01020916) [1] was a multicentre, multinational, outcome assessor-blinded, parallel group, randomised clinical trial comparing two strict target temperature regimens of 33°C and 36°C in adult patients, who had sustained return of spontaneous circulation and were unconscious after out-of-hospital cardiac arrest, when admitted to hospital. The trial did not demonstrate any difference in survival until end of trial (Hazard Ratio with a point estimate in favour of 36°C of 1.06 (95% confidence interval 0.89-1.28; P=0.51)) or neurologic function at six months after the arrest, measured with the Cerebral Performance Category (CPC) and the modified Rankin Scale (mRS).

The TTM2 trial is an international, multicentre, parallel group, non-commercial, randomised, superiority trial in which a target temperature of 33°C after cardiac arrest will be compared with normothermia and early treatment of fever (≥37.8°C)

Patients eligible for inclusion will be unconscious adult patients with out-of-hospital cardiac arrest of a presumed cardiac cause with stable return of spontaneous circulation. Randomisation will be performed by a healthcare professional in the emergency department, in the angiography suite or in the intensive care unit via web-based application using permuted blocks with varying sizes, stratified by site. Due to the nature of the intervention, health care staff will not be blinded to the intervention. However, outcome assessors, prognosticators, statisticians and conclusion drawers will be blinded to group allocation.

The intervention period will commence at the time of randomisation. Rapid cooling in the hypothermia group will be achieved by means of cold fluids and state-of-the-art cooling devices (intravascular/body-surface/nasal/oesophageal). A closed loop system will be used to maintain the target temperature. In the normothermia arm the aim will be early treatment of fever (≥37.8°C) using pharmacological measures and physical cooling when needed. For participants who develop a temperature of 37.8°C (trigger), a device will be used and set at 37.5°C. All participants will be sedated, mechanically ventilated and haemodynamically supported throughout the intervention period of 40 hours. At 28 hours after randomisation the participants in the hypothermia group will be rewarmed during 12 hours.

Participants who remain unconscious will be assessed according to a conservative protocol based on the European Resuscitation Council (ERC)'s recommendations for neurological prognostication after cardiac arrest.

Follow-up will be performed at 30 days, 6 and 24 months after cardiac arrest. The main results of the trial will be published following the 6-month follow-up, results from the long-term follow-up will be presented separately.

2 Background and Significance

In Europe approximately 300 000 inhabitants suffer an out-of-hospital cardiac arrest each year. Of those admitted to hospital with return of spontaneous circulation, the majority are unconscious and will need intensive care treatment and only 30-55% will be discharged alive. In survivors discharged from hospital the frequency of cognitive disability varies between reports. Using crude, but recommended, outcome scales such as the CPC-scale, Glasgow Outcome Scale (GOS), or the mRS, the general neurological function is good in the majority of patients, with only 10% having a severe neurological disability. In studies using more detailed instruments, cognitive impairment is reported to be present in 50% of survivors, and associated with lower quality of life and increased caregiver strain.

Many interventions have been tested in order to lower mortality and improve neurologic function in patients resuscitated after out-of-hospital cardiac arrest. Despite promising results in experimental models, all but one have failed in clinical trials. To date, induced hypothermia is the only intervention that has shown promising results in preliminary clinical trials.

2.1 Randomised Trials

In 2002 two small trials (n=77 and 275) reported a substantial improvement in survival and neurological function when unconscious patients with bystander witnessed out-of-hospital cardiac arrest (presumed cardiac origin and with initial shockable rhythms), were cooled to 32 to 34°C for 12 to 24 hours after return of spontaneous circulation. [2,3] These two trials received worldwide attention and international societies such as the American Heart Association (AHA), ERC, and the International Liaison Committee on Resuscitation (ILCOR) recommended the intervention in this patient group (strong recommendation, high level of evidence) and also for cardiac arrests of other origins, and with other initial rhythms. Cochrane reviews from 2009 and 2012 drew the same conclusion, strongly advocating hypothermia after cardiac arrest.

We performed a systematic review of the available evidence using meta-analysis, trial sequential analysis and the GRADE methodology and could report that earlier trials on hypothermia were at high risk of systematic error (bias), random errors (play of chance) and also hampered by obvious design errors [4] (for instance very selective inclusion criteria excluding more than 90 percent of potential patients). Our conclusion was that the overall quality of evidence was low, implying equipoise for additional research on hypothermia. In addition, it was clear that the optimal target temperature range was not defined and unclear whether the suggested benefit in earlier trials was attributable to hypothermia, or merely to avoiding the fever response, that is the natural trajectory for most unconscious cardiac arrest patients. We are currently writing a protocol for an updated version of our previous systematic review and review process will begin shortly.

With these findings in mind we designed and conducted the Targeted Temperature Management at 33°C versus 36°C after Cardiac Arrest trial during 26 months between 2010 and 2013. This trial included 950 participants in 36 hospitals in ten countries and randomised participants to 36 hours of temperature management at either 33°C or 36°C. The trial was more inclusive than earlier trials including 4 out of 5 unconscious patients with out-of-hospital cardiac arrest of a cardiac origin admitted to the emergency departments of the participating sites. The number of included participants was twice that of all previously randomised patients combined. The TTM1-trial did not demonstrate any difference in survival until end of trial (hazard ratio with a point

estimate in favour of 36°C of 1.06 (95% confidence interval 0.89-1.28; P=0.51)) or neurologic function at six months after the arrest, measured with CPC and mRS. Health-related quality-of-life did not differ significantly between the two groups. [5] Detailed cognitive testing in a large subset of patients detected cognitive impairment in approximately half of the surviving participants, with no difference between temperature groups. [6]

Critique of the trial has included that the hypothermia induction was not sufficiently rapid (although similar to previous trials), that the confidence limits were wide enough to include both clinically meaningful benefit and harm of the intervention, that subgroups within the general trial population could benefit from either intervention strategy, and that follow-up with neurocognitive testing should have been delayed further beyond the 6-month visit used in the trial. International guideline groups raised the following questions as a result of the TTM1-trial:

- Is fever control a sufficient measure to attenuate brain damage after cardiac arrest?
- Are there subgroups that would benefit from temperature management at a higher or lower level (for instance patients with longer arrests and more severe brain damage, or patients in circulatory shock)?
- Could faster and earlier induction of hypothermia improve outcomes in the 33°C-group?
- Were the results of the TTM1-trial not precise enough? Which would imply the need for larger sample sizes or meta-analytical approaches to better estimate effects.
- Could a longer follow-up perspective help in guiding which intervention is superior?

2.2 Hypothermia in other areas

In systematic reviews of multiple trials, hypothermia to 33°C was found effective in improving functional outcome in neonates with hypoxic ischaemic encephalopathy, a disease with many similarities with adult cardiac arrest. [7,8] In paediatric patients, one trial on in-hospital cardiac arrest was terminated early due to futility while a trial on out-of-hospital cardiac arrest showed no statistically significant difference between temperature groups. [9, 10] In contrast to this, a trial of hypothermia for adult traumatic brain injury showed consistently worse outcomes in the cooled group, and the trial was stopped early due to harm. [11]

2.3 Rationale for a new trial

The evidence for hypothermia in a broad context is conflicting. Clinical trials in various areas of brain damage indicate both benefit and harm. Theoretical rationale exists and currently hypothermia is the only neuroprotective strategy for cardiac arrest victims in clinical use. Specifically, in adults with cardiac arrest low quality evidence indicate benefit of 33°C and moderate quality evidence indicate no difference between 33°C and 36°C. The recent TTM1-trial has had a significant influence on the new ILCOR, AHA and ERC statements and guidelines for 2015, [12, 13] which have adopted the view that both lower and milder forms of temperature management provide similar clinical results. The recommended temperature range has been changed to include 36°C. Most importantly however, is that the overall evidence level for temperature management after out-of-hospital cardiac arrest has been changed to *low*, in line with our conclusion from the meta-analyses performed in 2010. In an international perspective, many hospitals and regions have already changed strategy in favour of the 36°C-arm, reasoning that

a less invasive and easier administrated temperature strategy yielding the same clinical results is preferable. Some hospitals however remain at 33°C based on earlier evidence while others, motivated by a lack of robust evidence, do not use temperature management at all.

Based on the above and the knowledge gaps indicated in international guidelines and reported by the Statens beredning för medicinsk och social utvärdering - Swedish agency for health technology assessment and assessment of social services (SBU), it is reasonable to assess whether rapidly administered hypothermia to a low target level is beneficial, and specifically to *a priori* define subgroups where the intervention effect could be studied. At the same time, it is important to clarify if early treatment of fever (easier, less costly and less invasive than the 36°C-arm in the TTM1-trial) is sufficient to achieve a good functional outcome. It is also important to, for the first time, investigate the evolution of neurological recovery over an extended period of time. We therefore propose the TTM2-trial.

2.4 Rationale for early treatment of fever

Fever is a risk factor for death after Cardiac arrest (CA) although it still remains an open question if it is a *causative and modifiable* risk factor. Zeiner and colleagues showed an increase in the odds of a poor neurological outcome for each degree higher than 37°C. [14] However, a body temperature above 37°C can occur due to individual or diurnal variation. When temperature is measured in a large population it appears that 37°C has no special significance to human thermometry. [15, 16] It therefore seems reasonable to apply a less strict definition of fever than >37.0°C. At the other end of the spectrum, it could be argued that it would be problematic to allow temperatures up to 38.3°C (A level usually employed in the definition of fever of unknown origin). [17]

This trial will employ normothermia-targeted temperature management in the control arm, with 37.8°C as a trigger for active temperature management with a feedback device. Although any temperature cut-off is to some extent arbitrary, the choice of these values is motivated by the following.

- Diagrammatic data from the HACA-trial [3] suggests a median temperature between 37.5°C and 37.8°C among patients in the control arm of the trial. If a similar distribution is assumed in the current trial a substantial number of patients will *not* require a device, thus making temperature management considerably less labour and resource intense.
- 37.7°C has been proposed as the upper limit of normal body temperature in healthy adults. [15] Employing active fever control for any patient who exceeds this temperature therefore constitutes an aggressive approach to fever control.
- Temperature fluctuations are unavoidable. In the TTM1-trial, the measured temperature among patients allocated to TTM at 36°C had a standard deviation of approximately 0.5°C. Assuming a similar variation around 37.5°C (for patients in whom active temperature management is used), few patients would become unequivocally febrile with temperatures above 38.3°C.

The functional definition of fever in this trial will therefore be temperatures greater than, or equal to 37.8°C. Normothermia will be defined as 36.5-37.7°C

We acknowledge that it is a limitation that the exact effects of fever control are unknown based on current evidence. We are currently writing two protocols with the titles:

A. Pharmacological fever control interventions for all conditions. A systematic review with meta-analysis and Trial Sequential Analysis

B. Non-pharmacological fever control interventions for all conditions. A systematic review with meta-analysis and Trial Sequential Analysis

The two reviews will contribute to a better understanding of the final results as the effects of the control-intervention will be better characterised.

3 Trial hypotheses and outcomes

Our objective will be to assess the beneficial and harmful effects of post-ischaemic hypothermia, when compared with normothermia and early treatment of fever in unconscious adults after out-of-hospital cardiac arrest.

3.1 Primary outcome

All-cause mortality assessed 180 days after randomisation

3.2 Secondary outcomes

- Proportion of patients with a poor functional outcome measured using the mRS-scale (mRS 0-3 vs 4-6) at 180 days after randomisation.
- Number of days alive and outside hospital within 180 days after randomisation (count data, days alive after first discharge from hospital within 180 days).
- Health-Related Quality of Life (HRQoL) using EQ5D-5L at 180 days after randomisation.
- Time-to-event (survival). All participants will be followed until the last included participant has been followed-up at 180 days. If death has not occurred, participants will be censored at this point.

3.3 Explorative outcomes

- Functional outcome measured using the mRS and Glasgow Outcome Scale-Extended (GOS-E) and mRS scales at 180 days after randomisation (ordinal data).
- Proportion of patients with a poor functional outcome measured using the GOS-E and mRS scales at 30 days after cardiac arrest.
- Neuro-cognitive function measured using the Montreal Cognitive Assessment (MoCA) and the Symbol Digit Modalities Test (SDMT). Neuro-cognitive function will be assessed at 180 days and at 24 months after randomisation.
- Self- and observer reported cognitive disability measured using Two Simple Questions (TSQ) and the Informant Questionnaire on Cognitive Decline in the Elderly (IQCODE). These tests will be performed at 180 days and at 24 months after randomisation.
- A repeat analysis of the primary outcome and all secondary outcomes at 24 months.

3.4 Rationale for chosen outcomes

To minimise biased assessment and to avoid competing risks, survival was chosen as the primary outcome. Although the intervention is primarily thought to affect the development of brain injury, survival is a global assessment of the intervention's effect on all organ systems. The estimated 45% mortality of the target population yields a high power to detect differences in a reasonably sized trial.

We recognise the risk that clinically relevant effects on the development of brain injury may be missed using survival as the only outcome, as neurological outcome for out-of-hospital cardiac arrest-survivors range from a vegetative state to complete recovery.

To complement and support the primary outcome we will therefore use the mRS-scale to evaluate functional outcome. [18–20] The mRS-scale is increasingly used in cardiac arrest, and is currently recommended in a ILCOR consensus statement as part of the Utstein template. [21] The scale will also likely be part of the core outcome set (COSCA) for cardiac arrest trials, which is being developed by an ILCOR consensus group including patient and partner representatives. To facilitate clinical interpretation of the trial results, and to provide an understandable effect size the primary analysis will be performed as a binary analysis, with the mRS-scale dichotomised (0-3 vs. 4-6).

To include patient reported outcome measures, HRQoL is recommended by guidelines for outcome reporting after cardiac arrest [22] and will likely be part of COSCA's recommendations. The EQ5D-5L was chosen as the TTM2-trial HRQoL-instrument since it is easy to use, validated, performs well when obtained by proxy and may be used to calculate quality-adjusted life-years. [23]

The GOS-E scale measures overall recovery and will be an explorative outcome in the TTM2-trial. GOS-E is an 8-point ordinal scale that has been validated for brain injury and reports effects on major life areas, ranging from levels of basic abilities (consciousness and dependence in everyday activities) to upper levels of a good recovery (return to a normal life, including work, and leisure activities). A standardised questionnaire and good psychometric properties secure reliable and valid outcome reports between multiple assessors and sites. [24] The commonly used CPC-scale can be extracted from the GOS-E to facilitate comparisons with other trials and and meta-analyses.

In the exploratory analyses we will use two tests to address the survivors' neuro-cognitive function in the domains mostly affected after CA: memory, executive functions and attention/mental processing speed. [22] The MoCA is a global cognitive screening test administered in approximately 10 minutes, which assesses multiple aspects of executive functions, short-term memory and delayed recall. [25] The SDMT is one of the most sensitive cognitive assessments to indicate brain injury and specifically assess attention/mental processing speed. [26] In a sub-study of the TTM1 trial the SDMT was the best discriminator of cognitive function between out-of-hospital cardiac arrest-patients and controls. [6] As in the TTM1-trial, we will use the 26-item IQCODE to obtain a relatives' perspective on changes in the participant's cognitive performance in everyday life [27] and the TSQ to obtain the patient reported cognitive outcome. [28] We have modified the IQCODE to the CA-situation. [29] The preliminary result of our validation study is that the psychometric properties are retained from the original test. In an attempt to measure a composite outcome of lower limb strength, proprioception and balance, the Times-Stand Test will also be performed. [30]

4 Eligibility

The trial population will be adults (18 years of age or older) who experience a non-traumatic cardiac arrest of a cardiac or unknown cause with return of spontaneous circulation (ROSC).

Patients will be eligible for enrolment if they meet all the following inclusion criteria and none of the exclusion criteria.

4.1 Inclusion criteria

A. Out-of-hospital cardiac arrest of a presumed cardiac or unknown cause

B. Sustained ROSC - defined as 20 minutes with signs of circulation without the need for chest compressions [31]

C. Unconsciousness defined as not being able to obey verbal commands (FOUR-score motor response of <4) and no verbal response to pain after sustained ROSC.

D. Eligible for intensive care without restrictions or limitations

E. Inclusion within 180 minutes of ROSC

4.2 Exclusion criteria

A. Unwitnessed cardiac arrest with an initial rhythm of asystole

B. Temperature on admission <30°C.

C. On ECMO prior to ROSC

D. Obvious or suspected pregnancy

E. Intracranial bleeding

F. Severe chronic obstructive pulmonary disorder (COPD) with long-term home oxygen therapy

4.3 Note on inclusion and exclusion criteria

In prior trials on hypothermia for cardiac arrest, inclusion criteria have usually included a cardiac or unknown cause of arrest. Since the update of the Utstein criteria [31] the term "medical cause of arrest" has been introduced. It is backward compatible with the earlier definition (presumed cardiac or unknown, other medical aetiologies). A medical cause of arrest can include asthma/COPD, anaphylaxis or GI-bleeding. We hypothesise that broadening the inclusion criteria would decrease the statistical power to detect a significant effect of the intervention because of an increased mortality due to other reasons than neurological damage in both temperature groups. The inclusion criteria of this trial have therefore not been edited to reflect this change in terminology.

There are three main reasons for including both participants with shockable and non-shockable rhythms. The first is that any neuroprotective effect of a lower target temperature reasonably would apply to both patient groups as the mechanism of cerebral injury is the same. Second,

it is reasonable to presume that any evidence for or against an intervention for patients with shockable rhythms will also be used for patients with non-shockable rhythm, as evidenced by the widespread use of hypothermia in both groups during the last decade. Third, including participants with non-shockable rhythms might increase the proportion of patients with a poor outcome, leading to an increased power.

Patients with refractory shock (systolic blood pressure <80mmHg despite receiving volume, inotropic/vasopressor support and/or an intra-aortic ballon pump (IABP)) will not be excluded from the trial. Results from the TTM1-trial showed that only 2% of patients assessed for eligibility in the trial were excluded due to refractory shock. The inclusion of these patients are therefore unlikely to effect the baseline risk of death in a significant way. Additionally, should the trial show positive results for hypothermia, the intervention will likely be used on patients in shock. We therefore deem the inclusion of these participants as a pragmatic approach.

Patients who are dependent on others for activities of daily living will not be excluded from the trial. Our experience from the TTM1-trial has been that a rapid ascertainment of the patient's pre-morbid functional status is difficult. To avoid any potential bias in recruitment, these patients will not be excluded. As the primary outcome will be death this will not impact the main results. Participants who are later identified to have had a pre-morbid status corresponding to mRS4-5 may be excluded from any analysis where participants are dichotomised into good and poor neurological outcomes.

Rather than gauging the patients' pre-morbid status before randomisation we think it more important to use factors that are known, and easier to establish from medical records at the moment of randomisation (limitations in care). The potential inclusion of a patient with a Do Not Resuscitate (DNR) order is likely to have a larger effect on the results than the inclusion of a patient with a pre-morbid mRS of 4.

4.4 Note on inclusion window

The inclusion window is from ROSC until 180 minutes after ROSC, however a patient is not eligible until stable ROSC (20 minutes without the need for CPR) has occurred. In practical terms, this means that the inclusion window is from 20 minutes after ROSC, until 180 minutes after ROSC.

In the event that a potential participant experiences sequential cardiac arrests, which is not uncommon, the inclusion window should be based on the state of consciousness and the presence of stable ROSC. The following scenarios might occur:

- A potential participant is resuscitated from an out-of-hospital cardiac arrest and is unconscious on admission to hospital. *Before 20 minutes* have passed, a second cardiac arrest occurs, after which the patient has stable ROSC. - This patient is eligible for inclusion as the second cardiac arrest is considered a continued event. Time to ROSC should be recorded as the time to stable ROSC (the second arrest) and the inclusion window starts at this time point.
- A potential participant is resuscitated from an out-of-hospital cardiac arrest and is unconscious on admission to hospital. *After 20 minutes* have passed, a second cardiac arrest occurs, after which the patient has stable ROSC. - This patient is eligible for inclusion. Time to ROSC should be recorded as the time to stable ROSC (the first arrest) and the inclusion window starts at this time point.

- A potential participant has an out-of-hospital cardiac arrest, is transported to hospital with ongoing CPR and ROSC occurs in the emergency room or angiography suite. - This patient is *eligible* for inclusion, as the initial event occurred outside the hospital walls.
- A potential participant has an out-of-hospital cardiac arrest and is conscious on admission to hospital. This is followed by a second cardiac arrest, stable ROSC, and unconsciousness. - This patient is *ineligible* as the event is considered an in-hospital arrest

4.5 Exit from the trial

A participant is free to withdraw his/her informed consent from the trial at any time after regaining consciousness. A participant will exit the trial if this participant withdraws consent. The reason for the exit will be collected and reported. The participant will be asked to specify which aspects of the trial he/she is withdrawing consent and participation from: attending the follow-up visits, diagnostic testing, inclusion of their data (including survival data) in a database, or publication. The participant making the withdrawal will be asked for permission to use data obtained prior to withdrawal and to obtain data for the primary outcome measure. If permission is obtained, the participant will be included in the final analyses. If the patient declines, all data from that patient will be destroyed.

If the trial intervention is discontinued by the treating physician because of adverse events, or any other reason, this does not constitute subject withdrawal from the trial and the patient will not exit the trial. All cases randomised in this trial will be analysed on an intention-to-treat basis.

5 Trial design

The TTM2-trial is a multicentre, international, randomised trial with a 1:1 concealed allocation. Participants with out-of-hospital cardiac arrest will be randomised to targeted temperature management with hypothermia at 33°C or normothermia and early treatment of fever equal to, or greater than 37.8°C. The trial will be investigator-initiated and non-commercial. Outcome assessors, prognosticators, statisticians, and conclusion drawers will be blinded to treatment allocation.

5.1 Screening and randomisation (Phase 1)

Screening can be performed either in the emergency room, angiography suite, or in the intensive care unit. Clinical investigators at each participating site will be responsible for screening of all patients who are resuscitated from an out-of-hospital cardiac arrest. A screening log will be compiled and include all out-of-hospital cardiac arrest-patients, whether they are eligible for inclusion, or not. Informed consent will be obtained according to national ethical approval.

Trial sites will have access to an internet based randomisation application to allow for immediate allocation and to ensure adequate allocation concealment and adequate generation of allocation sequence. Each patient will be assigned a unique trial and randomisation number. Randomisation will be performed with permuted blocks, stratified for trial site. *Phase 1* will be identical for both the intervention and control group.

5.2 Intervention period (Phase 2)

The intervention period will commence immediately after randomisation. Participants allocated to targeted temperature management at 33°C, will be rapidly cooled with a device. Upon reaching this temperature goal a maintenance phase will commence, which will end 28 hours after randomisation. During the maintenance phase the target temperature will be 33°C. This will be followed by rewarming at $\frac{1}{3}$°C/ hour. In the normothermia group the aim will be a temperature below 37.5°C. If conservative and pharmacological measures are insufficient and the temperature reaches 37.8°C, cooling with a device will be initiated with a target temperature of 37.5°C. All participants will be sedated and mechanically ventilated.

5.3 After the intervention period (Phase 3)

Extubation should be attempted at the earliest possible time, based on standard procedures for discontinuation of mechanical ventilation. For participants who remain in the ICU and are comatose or sedated at 40 hours after randomisation (end of the intervention) both allocation groups will have temperature maintained in the normal range with the aim to avoid fever until 72 hours after randomisation. Any use of a temperature management device in this phase will be at the discretion of the treating physician. Neurological evaluation will be performed by a blinded physician after a minimum of 96h have passed since randomisation.

5.4 General ICU-care

The general ICU-care is planned to be delivered similarly in both allocation groups according to local standardised care plans at the discretion of the treating physicians. Fluid therapy should be guided by standard procedures for haemodynamic support (fluid responsiveness, urinary output, haemodynamic and laboratory values, echocardiography etc). There will be treatment recommendations for sedation and management of shivering (outlined below). Management of haemodynamics, respiration, metabolic disturbances and seizures should be according to local protocols, at the discretion of the treating physician. Cardiac interventions will also be guided by local protocols, however participating centres will need to have access to around-the-clock invasive management, either on-site or at a nearby hospital also part of the trial. Cardiac catheterisation should not be delayed by the intervention, but efforts should be made to ensure temperature management during the procedure.

5.4.1 Sedation

Sedation will be mandatory for 40 hours after randomisation. There will not be a defined protocol for sedation and analgesia but short-acting drugs or volatile anaesthesia will be recommended. The sedative should be titrated to achieve deep sedation, a Richmond Agitation-Sedation Scale (RASS) of minus 4 should be targeted (No response to voice, but any movement to physical stimulation). [32]

Beyond adequate sedation during targeted temperature management, prolonged sedation is not recommended in international guidelines. Requiring 40 hours of sedation in both allocation arms therefore constitutes a departure from what is normally considered standard care. However, since participants included in the TTM2-trial would have received targeted temperature management and sedation, whether they were included in the trial or not, there is no difference in their treatment in regards to sedation. This approach is to facilitate a true comparison of two targeted temperatures, hypothermia and normothermia. Without sedation requirements there would be a substantial difference in the total dose of sedative agents between the hypothermia and normothermia treatment arms. Though the amount of sedatives administered in the hypothermia arm may still exceed the dose in the normothermia arm, a required sedation time is likely to lessen the difference.

5.4.2 Shivering

Shivering will be assessed according to the Bedside shivering assessment scale (BSAS). [33] The treatment goal for shivering will be to maintain a BSAS score of 0 or 1. To ensure adequate control of shivering the following protocol will be recommended. The recommended measures to reduce shivering should be continued through the entire intervention period in **both** allocation groups.

- Baseline care for all patients: Acetaminophen/Paracetamol administered either intravenously, parenterally or rectally, according to standard dosing guidelines. Acetaminophen/Paracetamol may be withheld at the discretion of the treating physician if liver dysfunction contraindicates the use. Buspirone, magnesium, clonidine, meperidine and skin counterwarming will be included in baseline care if these interventions are part of the local protocol for management of shivering. They will not be required for baseline care.

- Step 1: Increased sedation with propofol/dexmedetomidine and/or opiate. If the participant is haemodynamically unstable, midazolam may be substituted for propofol.
- Step 2: Administration of a neuromuscular blocking agent

5.4.3 The Bedside Shivering Assessment Scale (BSAS)

**0**	**None**	No shivering
**1**	**Mild**	Shivering localized to neck/thorax, may be seen only as artifact on ECG or felt by palpation
**2**	**Moderate**	Intermittent involvement of the upper extremities ±thorax
**3**	**Severe**	Generalized shivering or sustained upper/lower extremity shivering

5.5 Prognostication

Evaluating an intervention that cannot be blinded to the treating clinicians, the TTM2-trial will employ a conservative and strict protocol for neurological prognostication and related decisions regarding limitations in level-of-care - to mitigate potential bias.

Prognostication will be performed on *all* participants still in the ICU at 96 hours after randomisation. The prognostication will be based on the ERC and European Society for Intensive Care Medicine recommendations [12,34] and performed at approximately 96h after randomisation, but may be delayed due to practical reasons (such as weekend or national holiday). The physician performing the prognostication will be a neurologist, intensivist or other specialist experienced in neuroprognostication after cardiac arrest and who has not been involved in patient care. The prognosticator will be blinded for group allocation, but not for relevant clinical data. Prognostication and the potential decision to withdraw active intensive care are closely related but will be considered separate entities.

The result of the prognostication will be categorised as "YES" or "NO", based on the answer to the question *"Does this patient fulfil the TTM2-trial criteria for a likely poor neurological outcome?"*. This assessment will be recorded in the case report form and will be communicated to the treating clinician.

Any decision to withdraw active life support will be made by the treating physicians, together with the patient's relatives or legal surrogates, as required by local legislation. In making this decision the treating physician may use the information from the prognostication. The blinded external physician will not make any recommendation on WLST. Efforts will be made to sufficiently delay prognostication to ensure that any lingering effects of sedative agents will not affect the assessment.

Prognostication will be based on two mandatory, and four optional modalities.

5.5.1 Clinical examination - mandatory

A clinical examination including assessment of brainstem reflexes and described using the FOUR-score will be performed daily on all patients. An exception to the FOUR-score is that myoclonus will be considered separately in this trial. Absent or extensor motor response to pain (FOUR-score motor response 0-1) at 96h or later in a patient who is considered unaffected by sedative

agents, is a prerequisite to consider the neurologic prognosis poor. The bilateral absence of pupillary and corneal reflexes at 96h after CA or later, is a finding indicative of a poor prognosis.

The daily clinical examination by the ICU-staff should also include an assessment of status myoclonus (continuous and generalised myoclonus persisting for at least 30 min). A prospectively documented early status myoclonus (within 48 hours) is indicative of a poor prognosis.

Information from daily examinations including evaluation of status myoclonus should be available to the blinded physician performing the evaluation.

5.5.2 EEG - mandatory

An EEG performed between 48h and 96h after randomisation will be performed on all participants who survive, and remain unconscious to this point, in line with standard clinical practice. If it is not possible to perform an EEG study in the specified time frame due to practical reasons (such as weekend or national holiday), the EEG should be performed as soon as possible after 96h.

An EEG with a highly malignant pattern, and without reactivity to sound and pain is indicative of a poor prognosis.

5.5.3 Brain CT - optional

If a brain-CT shows signs of global ischaemic injury, such as: generalised oedema with reduced grey/white matter differentiation and sulcal effacement, this is indicative of a poor prognosis. A CT should be considered in patients who remain unconscious to exclude other pathologies such as intracranial haemorrhage or infarction.

5.5.4 Brain MRI - optional

A brain MRI at 3-5 days may be incorporated into prognostication if it has been performed. Signs of global, diffuse, or bilateral multifocal ischaemic lesions is indicative of a poor prognosis.

5.5.5 Neuron specific enolase- optional

High levels of Neuronspecific enolase (NSE) are indicative of a poor prognosis. NSE-sampling will not be mandatory, but may be used by sites with experience. If serial samples are available, and these are consistently higher than locally established levels associated with a poor outcome, this may be seen as indicative of a poor outcome. Samples with haemolysis should be disregarded.

5.5.6 SSEP - optional

Absent SSEP N20-responses bilaterally may be seen as indicative of a poor prognosis, if SSEP is performed more than 48h after randomisation.

5.5.7 The TTM2-trial criteria for a likely poor neurological outcome

In the TTM2 trial the prognosis is considered likely poor if criteria A, B and C are all fulfilled.

A Confounding factors such as severe metabolic derangement and lingering sedation has been ruled out.

B The patient has no response or a stereotypic extensor response to bilateral central and peripheral painful stimulation at ≥96 hours after randomisation.

C At least two of the below mentioned signs of a poor prognosis are present:

- **C1** Bilateral absence of pupillary and corneal reflexes at 96h after randomisation or later
- **C2** A prospectively documented early status myoclonus (within 48h of randomisation)
- **C3** A highly malignant EEG-pattern according to the TTM2 definition without reactivity to sound and painful stimulation. Patterns that are considered highly malignant are: [35, 36]
  - i. Suppressed background (amplitude <10mV, 100% of the recording) without discharges.
  - ii. Suppressed background with superimposed continuous periodic discharges.
  - iii. Burst-suppression (periods of suppression with amplitude <10mV constituting 50% of the recording) without discharges.
  - iv. Burst-suppression with superimposed discharges.
- **C4** CT brain with signs of global ischaemic injury, such as: generalised oedema with reduced grey/white matter differentiation and sulcal effacement or MRI-brain with signs of global, diffuse, or bilateral multifocal ischaemic lesions
- **C5** Serial serum-NSE samples consistently higher than locally established levels associated with a poor outcome
- **C6** Bilaterally Absent SSEP N20-responses more than 48 hours after randomisation

Note: *Participants with suspected ongoing status myoclonus at the time of assessment should still be assessed for a response to pain. An increase in the frequency or amplitude of myoclonic jerks when a painful stimuli is applied should not be considered as a motor response. If the participant localises to pain, the prognosis should not be stated as "likely poor neurological outcome", as this state may be compatible with a diagnosis of Lance-Adams syndrome.*

5.6 Withdrawal of life supporting therapies (WLST)

All participants in the trial will be actively treated until 96 hours after randomisation. There will be two exemptions from this rule.

- Participants in whom further treatment is considered unethical due to irreversible organ failure, a documented medical comorbidity, or other reasons
- Participants in whom brain death is established, however this will be defined as death and not WLST

The assumption of a poor neurological prognosis alone will not be considered sufficient to employ withdrawal of active intensive care prior to 96 hours after randomisation. After prognostication has been performed, WLST due to a presumed poor prognosis will be allowed if the TTM2-trial criteria for a likely poor neurological outcome are fulfilled and all effects of sedation on consciousness are ruled out.

Participants who have an unclear prognosis at 96h after randomisation should be reexamined daily and WLST may be considered if neurological function does not improve and, metabolic and pharmacological reasons for prolonged coma are ruled out. If a decision of WLST is made, the time point and the main reasons for withdrawing life-supporting therapies will be recorded. However supporting therapy may also be continued regardless of the neurological assessment of prognosis, at the discretion of the treating physician.

5.6.1 Brain death

Participants in whom brain death due to cerebral herniation is established will be registered as dead when a conclusive assessment has been made. If death is due to brain death this will be registered.

5.7 Follow up

A first formal follow-up will take place at 30 days after cardiac arrest. For some participants this follow-up will take place face-to-face in hospital. For those participants who are assessed after discharge, follow-up will be performed by telephone. Participants will be assessed according to the mRS-scale.

At six and twenty-four months, participants will be invited to a clinic visit, if possible with a relative or close friend. At these visits specially trained, blinded assessors will perform structured interviews and administer tests according to the secondary and exploratory outcomes. The assessment will focus on cognitive function, quality-of-life, return to work, participation in society cardiovascular risk factors including physical activity. At the twenty-four month visit participants will be approached for consent regarding a potential follow-up at 60 months.

The outcome-assessor may be an occupational therapist, physician, research nurse, psychologist or similar, who is proficient in the English language. Outcome-assessors will be provided with a written trial manual with detailed guidelines for performing the questionnaires and assessments. Training sessions will be provided by the trial coordinating team. At the end of each training session participants will perform mRS scoring on a number of practice cases. Outcome-assessors will also be encouraged to perform all follow-up procedures on a number of pilot persons.

5.8 Blinding

The clinical team responsible for the participant (physicians, nurses and others) and involved with direct patient care will not be blinded to allocation group due to the inherent difficulty in blinding the intervention and as temperature is a vital sign required for clinical care. Measures will be taken to ensure that the information about allocation will not disseminate beyond the immediate group of caregivers responsible for patient care. A blinded physician will evaluate the patient at 96 hours after randomisation and make a statement on neurological prognosis. The intensive care physician will not be allowed to share any information regarding temperature allocation group. Participants, their legal representatives, and family will only be informed that the patient has received targeted temperature management. Health personnel responsible for outcome assessment at follow-up will be blinded to the allocation of the intervention.

The steering group, author group, trial statistician, outcome assessors, prognosticators, statisticians and the trial coordinating team will be blinded to group allocation. The two intervention groups will be coded as "A" and "B". Two conclusions from all outcomes in the main manuscript will be drawn: one assuming "A" is the experimental group and "B" is the control group - and one assuming the opposite. All conclusion must be approved by the author group before the code is broken.

6 Coenrolment

In certain circumstances, coenrolment in multiple trials might be highly advantageous as research questions can be answered more quickly and data collection can be more efficient by collecting similar data. In other circumstances, coenrolment might be impossible due to logistical issues, or an interaction between the interventions.

It has been argued that coenrolment in RCTs should be assessed on a case-by-case basis in critical care as there are a wealth of methodological, legal and logistical issues that can arise when a patient is enrolled in more than one trial. [37] When assessing the possibility of coenrolment from a scientific standpoint, guidelines from the Canadian Critical Care group (CCCG) suggest that coenrolment of 1 ICU-patient into two RCTs only if:

- Interventions being tested in the 2 RCTs are commonly available interventions (e.g., recommendation against co-enrolment for new biologicals or devices for which mechanisms and outcomes are very uncertain)
- The intervention arms are unrelated and there is unlikely to be biologic interaction in the interventions tested in the 2 RCTs
- Both of the RCT Steering Committees are in agreement

6.1 Coenrolment in the TAME trial

The Targeted Therapeutic Mild Hypercapnia After Resuscitated Cardiac Arrest: A Phase III Multi-Centre Randomised Controlled Trial (TAME trial) (ACTRN12617000036314p) aims to determine whether targeted therapeutic mild hypercapnia improves neurological outcome at 6 months compared to standard care and targeted normocapnia. Coenrolment in this trial will be allowed, and encouraged for sites participating in the TTM2-trial. However, the option to participate in only the TTM2-trial will be retained for sites that are not willing, or able to coenroll. We consider coenrolment in TTM2 and TAME as an effective utilisation of research resources. We further consider that the methodological prerequisites suggested by the CCCG are fulfilled.

- Both interventions (temperature management and ability to apply TTMH) are readily available in all study ICUs.
- An interaction between TTM and TTMH is not likely. Though both interventions aim to reduce cerebral injury, the proposed mechanisms differ. While TTMH is proposed to improve cerebral blood flow, TTM is believed to have multiple neuroprotective effects including reductions in metabolic rate and pathologic cell signalling. We have studied the interaction between pCO_2 and temperature in the TTM-trial (unpublished data) and found no significant interaction. In an analysis which was adjusted for common predictors of outcome, participants with an average pCO_2 above 6kPa were not more likely to die (OR 1.03 (95%CI 0.45-2.37 , p=0.95). The interaction term between TTM at 36°C and an average pCO_2 of $\geq$6kPa was not significant (OR 0.49 (95%CI 0.13-1.77, p=0.27).
- The steering groups of the TTM2-Trial and the TAME-trial are in agreement regarding coenrolment. Inclusion criteria, data collection and protocols have been harmonised to facilitate coenrolment

There will be an appendix to this protocol outlining specific issues pertaining to co-enrolment between TTM2 and TAME. A letter of understanding between the two steering groups will also be appended.

6.1.1 Stratification

To avoid baseline imbalances in intervention arms, patients that are randomised in both TTM2 and TAME will be stratified according to allocation in the TAME trial (see separate statistical analysis plan).

6.2 Coenrolment in other trials

Study participants may be included in any observational trial which does not affect protocol adherence in the TTM2-trial. Pursuant to approval by the TTM2-steering committee, coenrolment in other randomised or intervention trials may be possible. Approval will be granted by the steering committee on a case-by-case basis.

7 Intervention period (Phase 2)

7.1 Hypothermia

Phase 2 of the trial starts immediately at randomisation. Temperature will be recorded hourly via a bladder thermometer. If the patient is oliguric, or if a bladder recording is not available the core temperature will be assessed by an oesophageal or intravascular probe. The total length of phase 2 will be 40 hours and will be divided into two blocks.

7.1.1 Block A - cooling phase and maintenance phase

Cooling can be induced by the following means:

- Intravenous cold (4°C) fluids. Normal saline, Hartman's solution or other similar crystalloids are recommended. The maximal volume for the initial cooling will be $30ml/kg^{-1}$.
- Approved endovascular cooling devices with closed loop systems
- Approved available surface cooling devices with closed loop systems
- Approved devices for intranasal or oesophageal cooling
- Ice-packs or cooling pads
- Pharmacological treatment with Acetaminophen/Paracetamol (part of anti-shivering protocol)
- Complete exposure of the patient
- Lowering of ambient temperature
- Any combination of the above that includes a device

After randomisation participants will be cooled as quickly as possible, with an initial target temperature of 33°C. The device used to induce hypothermia may be set at 32°C, to increase the speed of cooling. In the event that a device is used which does not allow a target temperature below 33°C, the device should be set at 33°C. If a patient has a temperature between 30°C and 33°C, the patient may be actively rewarmed to 33°C. However, passive rewarming between 30-33°C may also be used, if preferred by the treating physician.

The recommended method of cooling will by an approved and commercially available feedback controlled device (closed-loop device). To allow for a pragmatic trial, that does not limit the induction of hypothermia to any type of device and at the same time allow rapid cooling, initial cooling with cold fluids will be allowed. Use of neuromuscular blocking agents will be recommended to facilitate induction of hypothermia. Feasibility studies of novel cooling devices will not be allowed in the TTM2-trial.

The target temperature should be achieved as soon as possible. Local protocols should be developed to ensure that it is feasible to reach the targeted temperature within 90 minutes or shorter in the majority of participants.

When participants in the hypothermia arm reach $\leq$33°C the device setting should be immediately adjusted to 33°C.

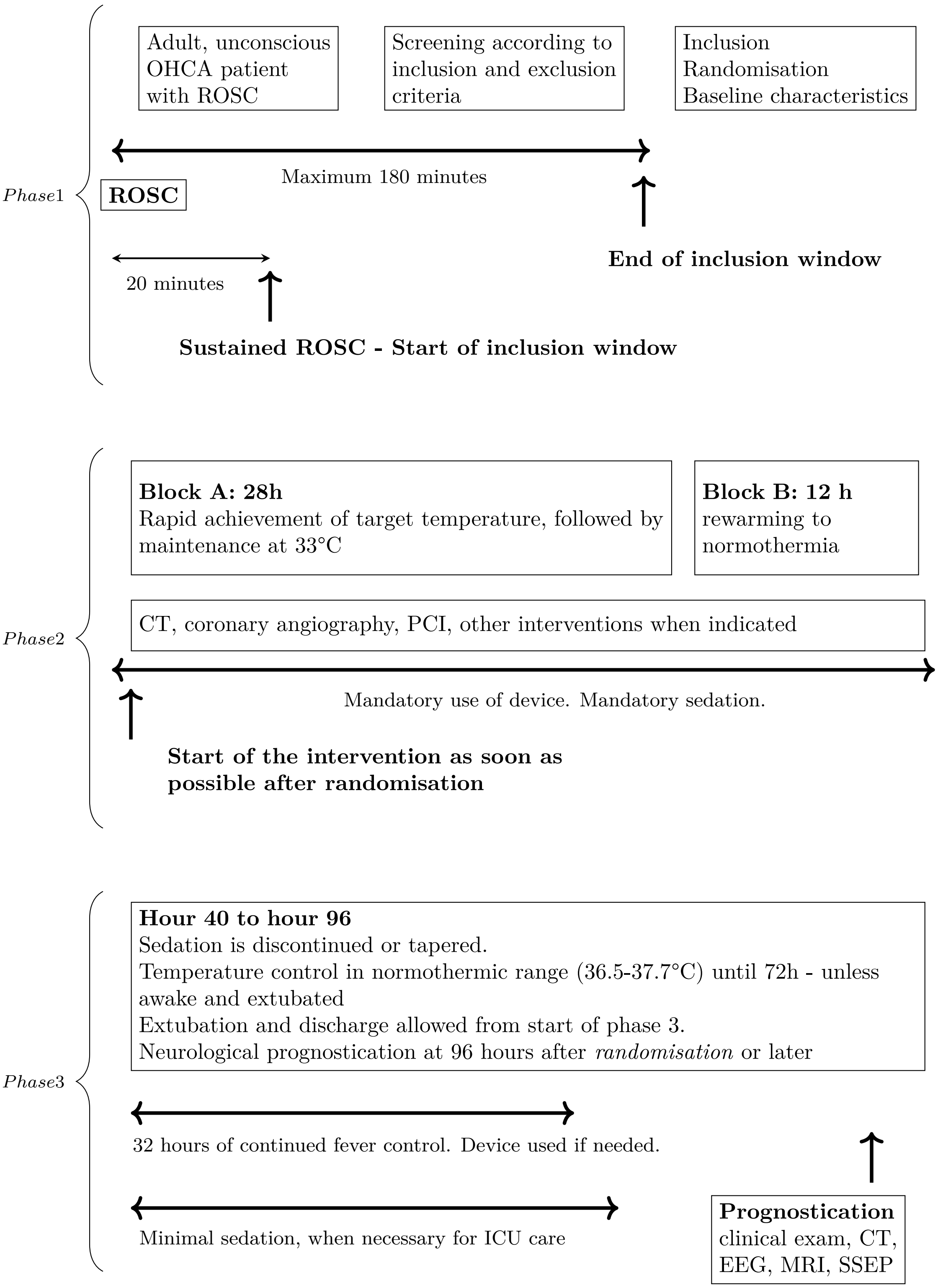

Figure 1: Schematic of trial intervention - Hypothermia

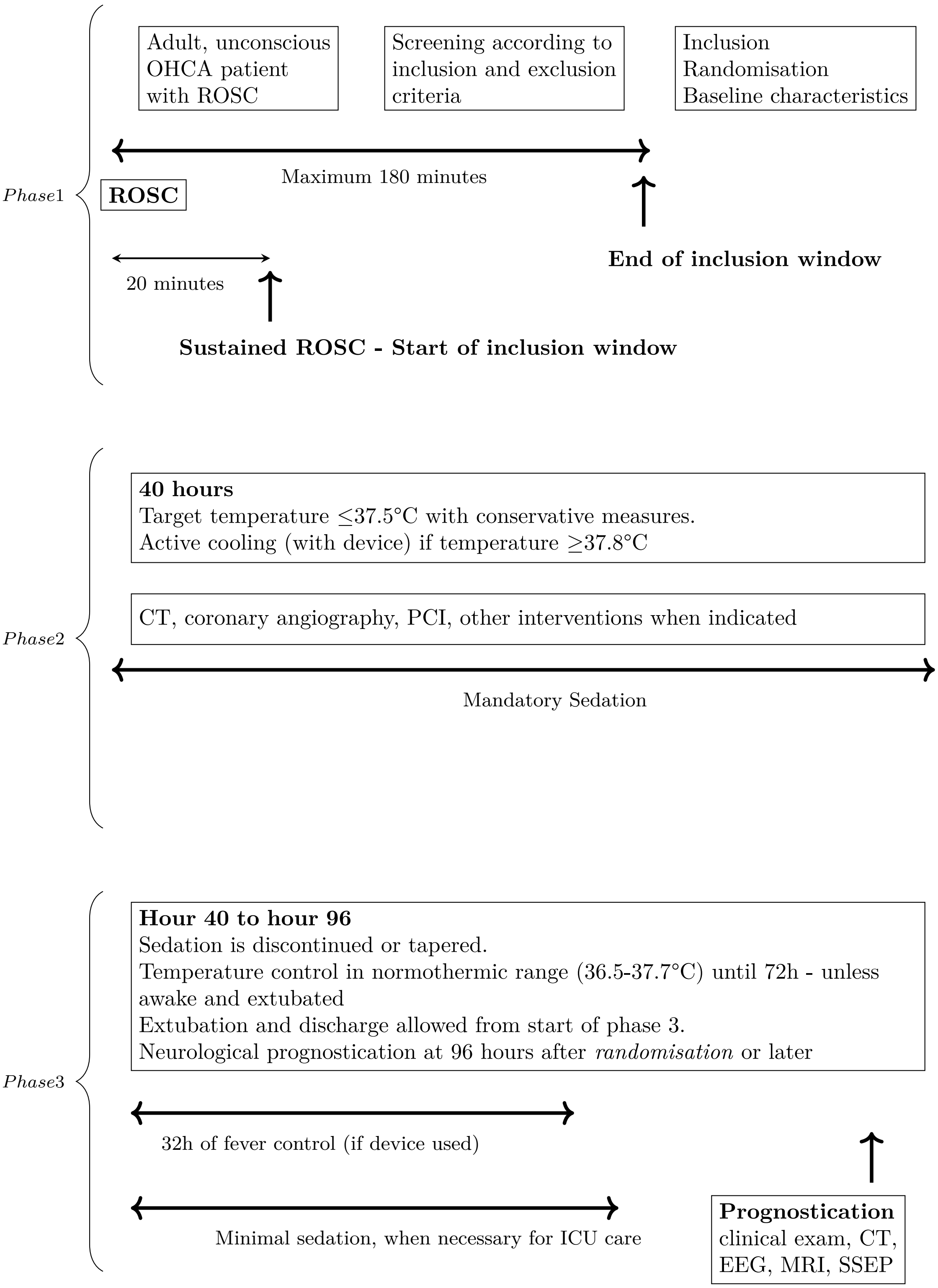

Figure 2: Schematic of control group - Normothermia and early treatment of fever

A target temperature and device setting of 33°C will be maintained until 28h after randomisation. Temperature control during the maintenance phase will be managed with an approved feedback controlled device.

7.1.2 Block B - Rewarming

Block B is the period (12h) allocated to restoration of normothermia. Participants will be rewarmed at $\frac{1}{3}$°C/ hour (1°C in three hours) allowing 12 hours for rewarming.

7.1.3 Early termination of the intervention

The intervention may be discontinued if hypothermia is the suspected cause of uncontrolled bleeding, life threatening arrhythmia or refractory haemodynamic instability, at the discretion of the treating physician. The target temperature will then be adjusted to ≤37.5°C

7.1.4 After rewarming

After 40 hours, those participants who remain comatose should be kept at a normothermic level (36.5 - 37.7°C) until 72h after randomisation and active warming should be avoided.

7.2 Normothermia and early treatment of fever

Normothermia, an active comparator will mirror the phases of the hypothermia intervention to ensure comparability between the allocation arms. However, the temperature management strategy will be different.

Temperature will be recorded via a bladder thermometer. If the patient is oliguric, or if a bladder recording is not available the core temperature will be assessed by an oesophageal or intravascular probe. Participants who have an initial temperature between 30-33°C may be actively rewarmed to 33°C, at which point active rewarming should be suspended. However, passive rewarming between 30-33°C may also be used, if preferred by the treating physician. Participants with an initial body temperature above 33°C will not be actively rewarmed to normothermia. To ensure that temperature does not reach 37.8°C the following conservative interventions will be allowed, at the discretion of the treating physician.

- Pharmacological treatment with Acetaminophen/Paracetamol (part of anti-shivering protocol)
- Complete exposure of the patient
- Lowering of ambient temperature

If conservative measures are insufficient, a device for temperature management will be used. The definition of insufficient fever control with conservative measures is:

A single recorded measurement of core body temperature ≥37.8°C, regardless whether the temperature is deemed to be of infectious origin or a response to neurological injury

If the criterion for insufficient fever control is fulfilled the same methods that will be used in the intervention arm will be used to achieve a target temperature of 37.5°C.

- Approved endovascular cooling devices with closed loop systems
- Approved available surface cooling devices with closed loop systems
- Intravenous cold (4°C) fluids for initial induction of hypothermia, if a device is not in situ. Normal saline, Hartman's solution or other similar crystalloids are recommended. The maximal volume will be 30ml/kg^{-1} or 2 liters. Fluids should be given whilst the device is being applied/inserted.

The treating physician may prescribe the application of a device (insert an endovascular catheter of apply a surface device) either prophylactically in all participants randomised to normothermia or if a rise in temperature is encountered. However the device will not be switched on until a core body temperature of ≥37.8°C is measured. Active fever control will be initiated as soon as a core body temperature reaches 37.8°C during the first 40 hours after randomisation. After 40 hours, those participants who remain comatose should be kept at a normothermic level (36.5 - 37.7°C) until 72h after randomisation and active warming should be avoided.

8 Data collection

Clinical, laboratory and background data will be collected at the time of enrolment, during the ICU-stay, at ICU-discharge, at hospital-discharge, and at follow-up. This section provides a summary of the data that will be collected.

Data will be obtained from hospital records, relatives, and ambulance services and will be entered into a web-based electronic case record form (eCRF) by site personnel. The site investigator must sign all eCRFs before trial completion to verify that the recorded data is correct and complete. The software for the web-based form will be provided by Spiral Web Solution Limited, New Zealand. Data from the web-based forms will be migrated to a trial database, which will be handled by the coordinating team.

The sponsor supplies a standard description of all units of measurement in the eCRF. If a trial site uses different units of measurement and this might be a potential source of error, the site investigator should contact the coordinating team to have the data capture module modified. Data not obtainable will be registered as missing and measures to obtain data should not delay intervention or concomitant treatment (i.e. central line not in place at the time of data collection)

8.1 Baseline data

This data will be obtained from emergency medical services/ambulance personnel or hospital records.

8.1.1 Pre-randomisation characteristics

- Inclusion and exclusion criteria
- Age
- Sex
- Time and date of ROSC
- Type of temperature management system planned (intravascular or surface cooling)

8.1.2 Pre-hospital data

- Scene of arrest (home, work, public place, nursing facility, other)
- Witnessed arrest (Y/N)
- Bystander CPR (Y/N)
- Bystander defibrillation performed (Y/N)
- First monitored rhythm at arrival of EMS
  (asystole, PEA, VF, non-perfusing VT, ROSC after bystander defibrillation, unknown (shockable or unshockable))

- Use of active compression-decompression device
  (No, Yes(LUCAS, Autopulse, manual))
- Number of defibrillations (if applicable)
- Date and time of cardiac arrest
- Time of emergency call
- Estimated time from arrest to advanced life support
- Pre-hospital airway (no, intubated, supra-glottic airway device)
- Amount of adrenaline (mg)

8.1.3 Background data

- Height
- Weight
- Pre-arrest neurological function
- Previous percutaneous coronary intervention? [Y/N]
- Previous coronary artery bypass grafting? [Y/N]
- Previous known cardiomyopathy? [Y/N]
- Previous implantable cardioverter defibrillator (ICD)? [Y/N]
- Previous atrial fibrillation of flutter? [Y/N]
- Previous hypertension with pharmacologic treatment? [Y/N]
- Charlson comorbidity index
- Measurement of pre-arrest frailty using the Clinical Frailty Score (1-9)

8.2 Data on hospital admission

- First recorded tympanic temperature (bilateral, highest value)
- FOUR-score
- STEMI - New ST-segment elevation $\geq$1 mm in $\geq$2 contiguous ECG leads
- ECG suspicious for acute ischaemia (No, ST-segment depressions (Y/N), T-wave inversions(Y/N), Acute LBBB(Y/N)
- ECG rhythm (sinus / atrial fibrillation or flutter / other)
- Prophylactic antibiotics prescribed (Y/N/Treatment warranted)
- SOFA score on admission (without neurological sub-score)
- Shock on admission, BP$<$90mmHg for at least 30 minutes or the need for supportive measure to maintain a systolic $\geq$90mmHg and end-organ hypoperfusion (cool extremities, or urine output of less than 30ml/hr, and a HR $>$60 beats per minute)

8.3 In the ICU

8.3.1 Data during the intervention

- Hourly temperature (bladder)
- Time (minutes) to core temperature of 34.0°C
- Mean arterial pressure, heart frequency
- Use of invasive haemodynamic monitoring (No, Thermodilution catheter, Pulmonary artery catheter)
- Cumulative doses of sedatives

8.3.2 Additional temperature measurements

- Between 41 and 48h after randomisation temperature will be recorded hourly
- Between 49 and 72h after randomisation temperature will be recorded every four hours

8.3.3 Daily during the ICU stay:

- FOUR-score, eye response, motor response, brainstem score, respiratory score
- SOFA score (without neurological sub-score)
- Need for mechanical circulatory assistance
- Highest body temperature
- Highest level of shivering (BSAS)
- Adverse events
- If trial intervention has been discontinued, time of discontinuation and specified reason
- If active intensive care is withdrawn, specify reason
- If dead, specify presumed cause of death, cardiac, cerebral, other

8.3.4 Neurology

- Results of the clinical neurological examination
- Results and time of SSEP, MRI, CT, NSE and EEG
- The stated prognosis from the blinded examiner, dichotomised as *poor outcome likely* (Y/N, based on the definition in the trial protocol) and which criteria are fulfilled

8.3.5 At ICU discharge

- Time and results of coronary angiography
- Time of PCI/open-heart surgery, if performed
- Discharge facility (coronary care unit/general ward/other ICU/dead)
- Results of microbial cultures for patients with sepsis

8.4 At hospital discharge

- Discharged to: nursing home/rehabilitation unit/other hospital/home/dead
  If dead, presumed cause of death: cardiac/cerebral/multiorgan failure/other
- Probable cause of cardiac arrest
- Organ donation (No/Donation after cardiac death/Donation after confirmed brain death)
- Autopsy performed (No/Yes)

8.5 30 days after randomisation

- If the patient is deceased, date of death, presumed cause of death: cardiac/cerebral/other
- Date of hospital discharge as obtained from hospital notes or registries
- mRS and GOS-E assessment by telephone interview

8.6 180 days after randomisation

- Survival status obtained from hospital or civil registries
- mRS and GOS-E assessment
- Cognitive function tested with MoCA, IQCODE, SDMT, Times-Stand Test, and health-related quality of life tested with: EQ5D-5L
- Cardiovascular risk factors (Physical activity, HbA1c, cholesterol, blood pressure)

8.7 24 months after randomisation

- Repeat evaluation of mRS, EQ5D-5L, GOS-E, cognitive tests, participation in society and cardiovascular risk factors, including physical activity

8.8 Planned investigations

Most investigations and interventions are performed at the discretion of the treating physician. However an EEG at 48-96h after randomisation is included in the protocol, for all patients who remain unconscious. Reasons for omission will be collected.

8.9 Laboratory testing

Laboratory testing will be performed as soon as possible after ROSC and continuously during the ICU-period. All blood gases will be analysed using the alpha-stat method.

- Earliest available blood gas after ROSC: FiO_2, pO_2,pCO_2, BE, pH, lactate, glucose.
- Blood gas every four hours during the intervention (FiO_2, pO_2, pCO_2, BE, pH, lactate, glucose and insulin dose)
- On admission to ICU (Lowest Thrombocytes, Lowest PaO_2, highest creatinine, highest bilirubin, HbA1c)
- Daily in the ICU (highest creatinine)

8.10 Biobank

Additional blood samples will be drawn at 0, 24, 48, and 72 hours after randomisation. Samples will be processed and aliquoted according to a separate protocol. All samples will be transported to, and stored in a central biobank. Blood samples may be analysed for routine clinical laboratory measurements and prognostic biomarkers, including markers of neuronal injury, inflammation and mitochondrial content. No analysis of nuclear DNA will be performed within the scope of the trial. No measurements will take place before the end of the trial, and no results from the biobank will be published in the initial manuscript.

8.10.1 Blood samples

- **0h:** serum vial 6 ml, plasma vial 6 ml, pax-RNA tube 2.5 ml
- **24h:** serum vial 6 ml
- **48h:** serum vial 6 ml, plasma vial 6 ml, pax-RNA tube 2.5 ml
- **72h:** serum vial 6 ml

9 Ethics and informed consent

An ethics application (2015/228) is approved by the Regional Ethics Committee at Lund University. Ethics applications will be submitted to all relevant ethics boards in every country participating. The ethics applications will seek approval for a delayed written consent process, since temperature management must be regarded as an emergency procedure and must be started as soon as the participants are admitted to the Emergency Departments. We judge that this strategy is justifiable according to the Declaration of Helsinki article 30 available from the World Medical Association. Participants regaining consciousness will be asked for written consent as soon as they are able to make an informed decision. The consenter will be provided with written and oral information on this trial to make an informed decision about participation in the trial. The consent form must be signed by the participant or legally acceptable surrogate and by the investigator seeking the consent. Relatives will be approached for written consent for their participation during follow-up visits.

10 Data management

10.1 Data handling and record keeping

Individual patient data will be handled as ordinary chart records and will be kept according to the legislation (e.g. data protection agencies) of each participating country. Data will be entered into the electronic database (eCRF) produced by Spiral Web Solution Limited, New Zealand. The electronic data capture module fulfils all criteria for handling of patient data according to the Swedish legislation on management of personal data "Personuppgiftslagen", (PUL) and is compliant with the Federal Drug Administration's guidelines for electronic signatures (FDA 21 CFR Part 11 Guidelines for Electronic Signatures). All original records (incl. consent forms, CRFs, SAE reports and relevant correspondence) will be retained at trial sites or the centre for Cardiac Arrest at Lund University for 15 years to allow inspection by relevant authorities. The trial database will be maintained for 15 years and anonymised if requested for revision.

10.2 Quality control and quality assurance

The trial will be externally monitored by national monitoring offices coordinated by the clinical trial manager and Clinical Studies Sweden, Forum South. The frequency of on-site monitoring will depend on compliance with the protocol, number of enrolled participants and data handling. At a minimum, there will be a pre-trial meeting, mandatory monitoring after the trial and once during the trial period. Source data verification will be performed according to a monitoring plan which will be available only to the trial monitors before the start of the trial.

All trial sites will be provided with sufficient information to participate in the trial. This document, CRFs, instructions for registration, checklists for inclusion/exclusion and randomisation, and a protocol for medical treatment will be distributed to all sites. Sites will also receive training on how to perform assessments at follow-up visits. The site investigator will be responsible for that all relevant data are entered into the electronic CRFs. The CRFs will be constructed in order to assure data quality with predefined values and ranges on all data entries. Data management activities will be performed and organised by the trial coordinating team.

11 Adverse events

Detection, documentation and reporting of the following events will be the responsibility of the local investigator.

11.1 Definitions

An **adverse event** is:

- Any untoward medical occurrence in a clinical trial subject

Untoward medial occurrences are expected in all patients who are resuscitated from cardiac arrest and treated in intensive care. This critically ill group of patients will per definition experience, be monitored and treated for untoward medical occurrences, and this is considered standard care. Therefore **no** adverse events will be reported.

A **Serious adverse event** is defined as any adverse event that:

- Results in death
- Is life threatening
- Requires hospitalisation or prolongation of current hospitalisation
- Results in persistent or significant disability or incapacity
- Results in a congenital anomaly/birth defect

Death is an expected outcome among survivors of cardiac arrest. Approximately 45% of patients will not survive to six months, therefore death will not be considered a serious adverse event. Standard care of cardiac arrest patients includes a host of complications that fit the definition of an SAE. For example, more than 90% of all patients in the TTM1-trial experienced a serious adverse event. Only a small number of those events could be considered unexpected or caused by the intervention. Additionally, when TTM at 33°C and 36°C was compared in the TTM1-trial, only hypokalaemia (which occurred in the majority of patients) differed between temperature groups.

The complications attributable to hypothermia in prior research primarily include electrolyte disorders, infection, arrhythmias, bleeding, haemodyanamic instability and skin complications related to the use of surface devices for temperature control. Despite this, none of the randomised trials on temperature control for cardiac arrest have shown any difference in the incidence of these complications (hypokalaemia in the TTM1-trial being the exception). To strike a balance between over-reporting, and maximise the probability of finding any true and important differences only the following will be considered serious adverse events:

Specific serious adverse events

- Pneumonia, defined as purulent tracheal secretions, a radiographic infiltrate and a decreased P/F ratio (PaO_2/FiO_2 <32 kPa or < 240 mmHg), a pragmatic adaption of the Clinical Pulmonary Infection Score (CPIS) [38]
- Sepsis and septic shock, according to the 3rd international consensus definitions for sepsis and septic shock [39]
- Moderate or severe bleeding, according to the GUSTO [40] criteria
- Device related skin complications (blistering or skin necrosis)
- Arrhythmias resulting haemodynamic compromise
- Bradycardia necessitating pacing

Other serious adverse events

- Unexpected serious adverse event

11.2 Reporting of serious adverse events

All serious adverse events not previously documented in the subject will be reported daily in the eCRF during the intensive care unit stay. Events that occur after discharge from the intensive care unit will not be reported. The specific serious adverse events described above will be reported whether they are considered related to the intervention or not. As the specific adverse events in many circumstances may be considered expected, they will not automatically mandate further follow-up.

At each daily assessment all unexpected serious adverse events either observed by the investigator or other caregivers must be recorded and evaluated. The event should be reported within 24 hours from awareness of the event using the eCRF. The nature and circumstances of the event should be described. Expected events is this population of participants include, but or not limited to haemodynamic instability, cardiac arrhythmias, electrolyte abnormalities, reintubation, worsening neurological function, cerebral oedema and complications related to the condition that led to cardiac arrest, and do not mandate reporting. An event which is considered expected in this population might still be considered unexpected in an individual participant. If this is the case, the event should be categorised as an unexpected serious adverse event and reported as such.

In the event that one of the specific adverse events occurs and the circumstances surrounding the event are unclear or unexpected it should be reported as an unexpected serious adverse event.

The relatedness between the trial intervention and the unexpected serious adverse event should be determined by the local investigator. The relatedness will be categorised as:

- **Not related:** The event is clearly related to other factors, such as the participant's clinical state, therapeutic interventions, or concomitant drugs administered to the participant
- **Possibly related:** There is a possible temporal relationship between the intervention and the event but it could have been caused by other factors

- **Probably related:** There is a plausible temporal relationship between the intervention and the event and the event is not reasonably explained by other factors.

The local investigator is required to follow each participant with an unexpected serious adverse event until resolution of symptoms. Reports of unexpected serious adverse event will be assessed for safety by a qualified physician in the trial coordinating team (medical monitor). The frequency of all serious adverse events (dichotomised by $\geq$1 event vs. no events) by will be reported to the DSMC.

12 Statistical plan and data analysis

A statistical analysis plan will be published before the first scheduled interim analysis.

12.1 Sample size

Based on the results of the TTM1-trial and information in the International cardiac arrest registry, (INTCAR) we estimate a total mortality of approximately 51.25%. The power calculation is based on a 55% mortality in the normothermia arm and a 47.5% mortality in the hypothermia arm, at 180 days.

To demonstrate a relative risk of 0.86 with 90% power at a significance level of 0.05, 931 participants are required in each group. The sample size calculation corresponds to a relative risk reduction (RRR) of 15%, an absolut risk reduction (ARR) of 7.5% and a number needed to treat (NNT) of 13.3. The estimated relative risk is based on results from earlier trials on hypothermia for CA. [2,3] To allow for a possible loss to follow-up we will recruit 1900 participants.

The analyses of the outcomes will be based on the intention-to-treat (ITT) principle, i.e., all randomised participants will be included in the analysis regardless of how much treatment they have received. Per-protocol analyses may be considered if important deviations from the protocol compromise the validity of the ITT analysis.

12.2 Analysis methods

All outcomes will initially be performed with adjustments made for site. Sensitivity analyses will be performed adjusting for a pre defined list of variables, which will include: site, age, sex, bystander CPR, initial rhythm, time to ROSC and circulatory status on admission.

12.2.1 Primary outcome

The primary outcome will be analysed as a binary variable (alive vs. dead) at 180 days.

12.2.2 Secondary outcome

Functional outcome will be evaluated by dichotomising the modified Rankin scale (0-3 vs 4-6). Survival data will be analysed using Cox regression. The secondary outcome HRQoL will primarily be presented as a the difference in the continuous VAS-scale included in the EQ5D-5L.

12.3 Missing data

Missing data will be reported in the publication. If further analyses reveals substantial missingness, multiple imputation will be considered.

12.4 Subgroup analysis

Subgroups will be analysed according to pre-defined variables

- Age
- Sex
- Bystander CPR
- Initial rhythm
- Time to ROSC
- Circulatory status on admission
- Severity classification, Pittsburgh cardiac arrest category [41]

12.5 Data safety monitoring committee

There will be an independent Data Safety Monitoring Committee (DSMC) arranging an independent statistician to conduct blinded interim analysis. The DSMC will be able to request unblinding of data if they find it necessary. The DSMC will be provided with data on survival and safety parameters continuously during the conduct of the trial, and can initiate analysis at any time they request. Lan-DeMets group sequential monitoring boundaries will be used if multiple interim analyses are needed. The DSMC may stop or pause the trial if:

- Group difference in the primary outcome measure is found in the interim analysis according to pre-defined stopping rules
- Group difference in serious adverse events is found in the interim analysis
- Results from other studies show benefit or harm with one of the allocation arms

12.5.1 Members of the DSMC-committee

**Kathy Rowan**	Intensive Care National Audit & Research Centre, UK (Chair)
**David Harrison**	Intensive Care National Audit & Research Centre, UK
**Paul Mouncey**	Intensive Care National Audit & Research Centre, UK
**Duncan Young**	Nuffield Department of Clinical Neurosciences, University of Oxford, UK
**Manu Shankar-Hari**	Guy's and St Thomas's NHS Foundation Trust, London, UK

13 Publication of Data

The trial will be analysed by two independent statisticians and the results interpreted by the steering group. The analysis will be performed 28 weeks after inclusion of the last patient. The analysis process will be performed with the allocation code unbroken and with the trial arms only known as A and B. Two abstracts will be prepared before the allocation code is broken, with the different arms inter-changed (one assuming arm A is hypothermia, and the other assuming arm B is hypothermia). All authors must approve both versions before the code is broken. The final manuscript will be submitted to a peer-reviewed international journal. Authorship will be granted using the Vancouver definitions and depending on personal involvement and fulfilment of the author's respective roles. The author list will include the steering group members, national investigators and additional names. Centres recruiting >30 participants will be entitled to one name, >60 two names, >100 three names, >150 four names, >220 five names in the author list (additional names). After the author list there will be added: "and the TTM-trial group" and a reference to an appendix with all sites, site investigators and number of participants enrolled. The main publication will report the primary and secondary outcomes. In doing so, survival, functional outcome and HRQoL will be reported. Exploratory outcomes will, due to complexity of reporting be submitted to a peer-reviewed journal as a separate manuscript, as will the results from the 24 month follow-up. A detailed authorship plan will be decided upon after the first interim analysis.

14 Insurance

When preexisting insurance is not available, indemnity to meet the potential legal liability of investigators/collaborating hospitals for harm to participants arising from the conduct of the research will be provided by the TTM2-Trial through the sponsor: Region Skåne - Skånevård SUND. The insurance negotiated with Allianz insurance company for each country will be specified in each site agreement before the commencement of patient inclusion at that site.

15 Funding

The trial will be funded by external foundations for medical research. Patient recruitment will not commence until there is sufficient funding to allow for inclusion and 180-day follow-up of the proposed sample size.
The trial is funded by:

- The Swedish Research Council(Vetenskapsrådet) - Grant Nr: 2016-00428
- The Swedish Heart-Lung Foundation
- Stig and Ragna Gorthon Foundation
- Knutsson Foundation

16 Timeline

**2016**	Trial design, ethics application, site recruitment, application for funding
**2017**	First patient recruitment, run-in period, site initiations
**2017-2019**	Patient recruitment and interim analysis
**2019/2020**	Presentation of results, long-term follow-up performed
**2022**	Presentation of long-term outcomes

17 Trial Participants

17.1 Steering Group

**Niklas Nielsen** MD, PhD	Intensive Care, Helsingborg Hospital, Helsingborg, Sweden (CI, Study Chair)
**Jan Bělohlávek** MD, PhD	General University Hospital, Prague,Czech Republic (NI)
**Clifton Callaway** MD, PhD	Emergency medicine, University of Pittsburgh, Pittsburgh, USA (NI)
**Alain Cariou** MD, PhD	Intensive Care, Descartes University, Paris, France (NI)
**Tobias Cronberg** MD, PhD	Neurology, Skåne University Hospital, Lund, Sweden (SI)
**Josef Dankiewicz** MD, PhD	Cardiology, Skåne University Hospital, Lund, Sweden (CoI)
**David Erlinge** MD, PhD	Cardiology, Skåne University Hospital, Lund, Sweden
**Hans Friberg** MD, PhD	Intensive Care, Skåne University Hospital, Malmö, Sweden (SI)
**Jan Hovdenes** MD, PhD	Intensive Care, Rikshospitalet, Oslo University Hospital, Oslo, Norway (NI)
**Janus Christian Jakobsen** MD, PhD	Copenhagen Trial Unit, Copenhagen University Hospital, Copenhagen, Denmark (Trialist)
**Michael Joannidis** MD, PhD	Intensive Care, Medical University Innsbruck, Austria (NI)
**Michael Kuiper** MD, PhD	Intensive Care, Leeuwarden Hospital, Leeuwarden, The Netherlands
**Helena Levin** MSc	Centre for Cardiac Arrest, Lund, Sweden (Clinical trial manager)
**Gisela Lilja** OT, PhD	Neurology and rehabilitation medicine, Lund, Sweden (follow-up coordinator)
**Per Nordberg** MD, PhD	Cardiology, Södersjukhuset, Stockholm, Sweden
**Mauro Oddo** MD, PhD	Intensive Care, Université de Lausanne, Lausanne, Switzerland (NI)
**Paolo Pelosi**, MD, FERS	Anaesthesia and Intensive Care - IRCCS AOU San Martino IST, University of Genova, Genova, Italy (NI)
**Christian Rylander** MD, PhD	Sahlgrenska University Hospital, Gothenburg, Sweden (NI)
**Manoj Saxena** MD, PhD	Intensive Care, The George Institute for Global Health, Sydney, Australia (NI)
**Pascal Stammet** MD, PhD	Centre Hospitalier de Luxembourg, Luxembourg (NI)
**Christian Storm** MD, PhD	Chartité University Hospitals, Berlin, Germany (NI)
**Fabio Taccone** MD, PhD	Hopital Erasme, Brussles, Belgium (NI)
**Susann Ullén** PhD	Clinical trials Sweden - Forum South, Lund, Sweden (Chief Statistician)
**Matthew P. Wise** MD, DPhil	University Hospital of Wales, Cardiff, UK (NI)

CI - Chief Investigator
SI - Senior Investigator
NI - National Investigator
CoI - Coordinating investigator

17.2 Investigators - TBD

17.3 Investigator responsibilities

The trial site investigator is responsible for:

- Screening and listing eligible patients
- Performing randomisation
- Achieving temperature control according to allocation group
- Ensuring that achievement of hypothermia is feasible within 2 hours of randomisation
- Maintaining temperature control according to allocation group
- Collection and reporting of data according to the trial protocol and electronic Case Report Form (eCRF)
- Obtaining written informed consent from patients whom regain consciousness
- Performing and reporting follow-up according to the trial protocol and the eCRF

The national investigator is responsible for:

- Coordination of national sites
- Representing national sites in the steering group
- Reviewing reasons for potential incomplete screening and randomisation at national sites
- Ethical Review Board - application and approval
- Dissemination of protocols and updates to sites
- Proposing suitable candidates for vacant site investigator positions

References

[1] Niklas Nielsen, Jørn Wetterslev, Tobias Cronberg, David Erlinge, Yvan Gasche, Christian Hassager, Janneke Horn, Jan Hovdenes, Jesper Kjaergaard, Michael Kuiper, Tommaso Pellis, Pascal Stammet, Michael Wanscher, Matt P Wise, Anders Åneman, Nawaf Al-Subaie, Søren Boesgaard, John Bro-Jeppesen, Iole Brunetti, Jan Frederik Bugge, Christopher D Hingston, Nicole P Juffermans, Matty Koopmans, Lars Køber, Jørund Langørgen, Gisela Lilja, Jacob Eifer Møller, Malin Rundgren, Christian Rylander, Ondrej Smid, Christophe Werer, Per Winkel, Hans Friberg, and TTM Trial Investigators. Targeted temperature management at 33 °c versus 36 °c after cardiac arrest. *N Engl J Med*, 369(23):2197–206, Dec 2013.

[2] Stephen A Bernard, Timothy W Gray, Michael D Buist, Bruce M Jones, William Silvester, Geoff Gutteridge, and Karen Smith. Treatment of comatose survivors of out-of-hospital cardiac arrest with induced hypothermia. *N Engl J Med*, 346(8):557–63, Feb 2002.

[3] Hypothermia after Cardiac Arrest Study Group. Mild therapeutic hypothermia to improve the neurologic outcome after cardiac arrest. *N Engl J Med*, 346(8):549–56, Feb 2002.

[4] Silvio Garattini, Janus C Jakobsen, Jørn Wetterslev, Vittorio Bertelé, Rita Banzi, Ana Rath, Edmund A M Neugebauer, Martine Laville, Yvonne Masson, Virginie Hivert, Michaela Eikermann, Burc Aydin, Sandra Ngwabyt, Cecilia Martinho, Chiara Gerardi, Cezary A Szmigielski, Jacques Demotes-Mainard, and Christian Gluud. Evidence-based clinical practice: Overview of threats to the validity of evidence and how to minimise them. *Eur J Intern Med*, 32:13–21, Jul 2016.

[5] Tobias Cronberg, Gisela Lilja, Janneke Horn, Jesper Kjaergaard, Matt P Wise, Tommaso Pellis, Jan Hovdenes, Yvan Gasche, Anders Åneman, Pascal Stammet, David Erlinge, Hans Friberg, Christian Hassager, Michael Kuiper, Michael Wanscher, Frank Bosch, Julius Cranshaw, Gian-Reto Kleger, Stefan Persson, Johan Undén, Andrew Walden, Per Winkel, Jørn Wetterslev, Niklas Nielsen, and TTM Trial Investigators. Neurologic function and health-related quality of life in patients following targeted temperature management at 33 °c vs 36 °c after out-of-hospital cardiac arrest: A randomized clinical trial. *JAMA Neurol*, 72(6):634–41, Jun 2015.

[6] Gisela Lilja, Niklas Nielsen, Hans Friberg, Janneke Horn, Jesper Kjaergaard, Fredrik Nilsson, Tommaso Pellis, Jørn Wetterslev, Matt P Wise, Frank Bosch, John Bro-Jeppesen, Iole Brunetti, Azul Forti Buratti, Christian Hassager, Caisa Hofgren, Angelo Insorsi, Michael Kuiper, Alice Martini, Nicki Palmer, Malin Rundgren, Christian Rylander, Annelou van der Veen, Michael Wanscher, Helen Watkins, and Tobias Cronberg. Cognitive function in survivors of out-of-hospital cardiac arrest after target temperature management at 33 °c versus 36 °c. *Circulation*, 131(15):1340–9, Apr 2015.

[7] Peter D Gluckman, John S Wyatt, Denis Azzopardi, Roberta Ballard, A David Edwards, Donna M Ferriero, Richard A Polin, Charlene M Robertson, Marianne Thoresen, Andrew Whitelaw, and Alistair J Gunn. Selective head cooling with mild systemic hypothermia after neonatal encephalopathy: multicentre randomised trial. *Lancet*, 365(9460):663–70, 2005.

[8] Seetha Shankaran, Abbot R Laptook, Richard A Ehrenkranz, Jon E Tyson, Scott A McDonald, Edward F Donovan, Avroy A Fanaroff, W Kenneth Poole, Linda L Wright, Rosemary D Higgins, Neil N Finer, Waldemar A Carlo, Shahnaz Duara, William Oh, C Michael Cotten, David K Stevenson, Barbara J Stoll, James A Lemons, Ronnie Guillet, Alan H Jobe, and

National Institute of Child Health and Human Development Neonatal Research Network. Whole-body hypothermia for neonates with hypoxic-ischemic encephalopathy. *N Engl J Med*, 353(15):1574–84, Oct 2005.

[9] Frank W Moler, Faye S Silverstein, Richard Holubkov, Beth S Slomine, James R Christensen, Vinay M Nadkarni, Kathleen L Meert, Brittan Browning, Victoria L Pemberton, Kent Page, Marianne R Gildea, Barnaby R Scholefield, Seetha Shankaran, Jamie S Hutchison, John T Berger, George Ofori-Amanfo, Christopher J L Newth, Alexis Topjian, Kimberly S Bennett, Joshua D Koch, Nga Pham, Nikhil K Chanani, Jose A Pineda, Rick Harrison, Heidi J Dalton, Jeffrey Alten, Charles L Schleien, Denise M Goodman, Jerry J Zimmerman, Utpal S Bhalala, Adam J Schwarz, Melissa B Porter, Samir Shah, Ericka L Fink, Patrick McQuillen, Theodore Wu, Sophie Skellett, Neal J Thomas, Jeffrey E Nowak, Paul B Baines, John Pappachan, Mudit Mathur, Eric Lloyd, Elise W van der Jagt, Emily L Dobyns, Michael T Meyer, Ronald C Sanders, Jr, Amy E Clark, J Michael Dean, and THAPCA Trial Investigators. Therapeutic hypothermia after in-hospital cardiac arrest in children. *N Engl J Med*, 376(4):318–329, 01 2017.

[10] Frank W Moler, Faye S Silverstein, Richard Holubkov, Beth S Slomine, James R Christensen, Vinay M Nadkarni, Kathleen L Meert, Amy E Clark, Brittan Browning, Victoria L Pemberton, Kent Page, Seetha Shankaran, Jamie S Hutchison, Christopher J L Newth, Kimberly S Bennett, John T Berger, Alexis Topjian, Jose A Pineda, Joshua D Koch, Charles L Schleien, Heidi J Dalton, George Ofori-Amanfo, Denise M Goodman, Ericka L Fink, Patrick McQuillen, Jerry J Zimmerman, Neal J Thomas, Elise W van der Jagt, Melissa B Porter, Michael T Meyer, Rick Harrison, Nga Pham, Adam J Schwarz, Jeffrey E Nowak, Jeffrey Alten, Derek S Wheeler, Utpal S Bhalala, Karen Lidsky, Eric Lloyd, Mudit Mathur, Samir Shah, Theodore Wu, Andreas A Theodorou, Ronald C Sanders, Jr, J Michael Dean, and THAPCA Trial Investigators. Therapeutic hypothermia after out-of-hospital cardiac arrest in children. *N Engl J Med*, 372(20):1898–908, May 2015.

[11] Peter J D Andrews, H Louise Sinclair, Aryelly Rodriguez, Bridget A Harris, Claire G Battison, Jonathan K J Rhodes, Gordon D Murray, and Eurotherm3235 Trial Collaborators. Hypothermia for intracranial hypertension after traumatic brain injury. *N Engl J Med*, 373(25):2403–12, Dec 2015.

[12] Jerry P Nolan, Jasmeet Soar, Alain Cariou, Tobias Cronberg, Véronique R M Moulaert, Charles D Deakin, Bernd W Bottiger, Hans Friberg, Kjetil Sunde, and Claudio Sandroni. European resuscitation council and european society of intensive care medicine guidelines for post-resuscitation care 2015: Section 5 of the european resuscitation council guidelines for resuscitation 2015. *Resuscitation*, 95:202–22, Oct 2015.

[13] Clifton W Callaway, Michael W Donnino, Ericka L Fink, Romergryko G Geocadin, Eyal Golan, Karl B Kern, Marion Leary, William J Meurer, Mary Ann Peberdy, Trevonne M Thompson, and Janice L Zimmerman. Part 8: Post-cardiac arrest care: 2015 american heart association guidelines update for cardiopulmonary resuscitation and emergency cardiovascular care. *Circulation*, 132(18 Suppl 2):S465–82, Nov 2015.

[14] A Zeiner, M Holzer, F Sterz, W Schörkhuber, P Eisenburger, C Havel, A Kliegel, and A N Laggner. Hyperthermia after cardiac arrest is associated with an unfavorable neurologic outcome. *Arch Intern Med*, 161(16):2007–12, Sep 2001.

[15] P A Mackowiak, S S Wasserman, and M M Levine. A critical appraisal of 98.6 degrees f, the upper limit of the normal body temperature, and other legacies of carl reinhold august

wunderlich. *JAMA*, 268(12):1578–80, 1992.

[16] Märtha Sund-Levander, Christina Forsberg, and Lis Karin Wahren. Normal oral, rectal, tympanic and axillary body temperature in adult men and women: a systematic literature review. *Scand J Caring Sci*, 16(2):122–8, Jun 2002.

[17] R G PETERSDORF and P B BEESON. Fever of unexplained origin: report on 100 cases. *Medicine (Baltimore)*, 40:1–30, Feb 1961.

[18] J RANKIN. Cerebral vascular accidents in patients over the age of 60. ii. prognosis. *Scott Med J*, 2(5):200–15, May 1957.

[19] B Farrell, J Godwin, S Richards, and C Warlow. The united kingdom transient ischaemic attack (uk-tia) aspirin trial: final results. *J Neurol Neurosurg Psychiatry*, 54(12):1044–54, Dec 1991.

[20] J C van Swieten, P J Koudstaal, M C Visser, H J Schouten, and J van Gijn. Interobserver agreement for the assessment of handicap in stroke patients. *Stroke*, 19(5):604–7, May 1988.

[21] Gavin D Perkins, Ian G Jacobs, Vinay M Nadkarni, Robert A Berg, Farhan Bhanji, Dominique Biarent, Leo L Bossaert, Stephen J Brett, Douglas Chamberlain, Allan R de Caen, Charles D Deakin, Judith C Finn, Jan-Thorsten Gräsner, Mary Fran Hazinski, Taku Iwami, Rudolph W Koster, Swee Han Lim, Matthew Huei-Ming Ma, Bryan F McNally, Peter T Morley, Laurie J Morrison, Koenraad G Monsieurs, William Montgomery, Graham Nichol, Kazuo Okada, Marcus Eng Hock Ong, Andrew H Travers, Jerry P Nolan, and Utstein Collaborators. Cardiac arrest and cardiopulmonary resuscitation outcome reports: update of the utstein resuscitation registry templates for out-of-hospital cardiac arrest: a statement for healthcare professionals from a task force of the international liaison committee on resuscitation (american heart association, european resuscitation council, australian and new zealand council on resuscitation, heart and stroke foundation of canada, interamerican heart foundation, resuscitation council of southern africa, resuscitation council of asia); and the american heart association emergency cardiovascular care committee and the council on cardiopulmonary, critical care, perioperative and resuscitation. *Circulation*, 132(13):1286–300, Sep 2015.

[22] Lance B Becker, Tom P Aufderheide, Romergryko G Geocadin, Clifton W Callaway, Ronald M Lazar, Michael W Donnino, Vinay M Nadkarni, Benjamin S Abella, Christophe Adrie, Robert A Berg, Raina M Merchant, Robert E O'Connor, David O Meltzer, Margo B Holm, William T Longstreth, Henry R Halperin, American Heart Association Emergency Cardiovascular Care Committee, and Council on Cardiopulmonary, Critical Care, Perioperative and Resuscitation. Primary outcomes for resuscitation science studies: a consensus statement from the american heart association. *Circulation*, 124(19):2158–77, Nov 2011.

[23] M Herdman, Claire Gudex, A Lloyd, MF Janssen, P Kind, D Parkin, Gouke Bonsel, and Xavier Badia. Development and preliminary testing of the new five-level version of eq-5d (eq-5d-5l). *Quality of life research*, 20(10):1727–1736, 2011.

[24] J T Wilson, L E Pettigrew, and G M Teasdale. Structured interviews for the glasgow outcome scale and the extended glasgow outcome scale: guidelines for their use. *J Neurotrauma*, 15(8):573–85, Aug 1998.

[25] Ziad S Nasreddine, Natalie A Phillips, Valérie Bédirian, Simon Charbonneau, Victor Whitehead, Isabelle Collin, Jeffrey L Cummings, and Howard Chertkow. The montreal cognitive

assessment, moca: a brief screening tool for mild cognitive impairment. *J Am Geriatr Soc*, 53(4):695–9, Apr 2005.

[26] A. Smith. *A. Smith Symbol Digits Modalities Test Western Psychological Services, Los Angeles (1982).* Western Psychological Services, Los Angeles, 1982.

[27] AF Jorm and PA Jacomb. The informant questionnaire on cognitive decline in the elderly (iqcode): socio-demographic correlates, reliability, validity and some norms. *Psychol Med*, 19(4):1015–1022, 1989.

[28] WT Longstreth, Graham Nichol, Lois Van Ottingham, and Alfred P Hallstrom. Two simple questions to assess neurologic outcomes at 3 months after out-of-hospital cardiac arrest: experience from the public access defibrillation trial. *Resuscitation*, 81(5):530–533, 2010.

[29] Gisela Lilja, Niklas Nielsen, Hans Friberg, Janneke Horn, Jesper Kjaergaard, Tommaso Pellis, Malin Rundgren, Jørn Wetterslev, Matt P Wise, Fredrik Nilsson, et al. Cognitive function after cardiac arrest and temperature management; rationale and description of a sub-study in the target temperature management trial. *BMC cardiovascular disorders*, 13(1):1, 2013.

[30] Stephen R Lord, Susan M Murray, Kirsten Chapman, Bridget Munro, and Anne Tiedemann. Sit-to-stand performance depends on sensation, speed, balance, and psychological status in addition to strength in older people. *J Gerontol A Biol Sci Med Sci*, 57(8):M539–43, Aug 2002.

[31] I Jacobs, V Nadkarni, J Bahr, RA Berg, JE Billi, L Bossaert, P Cassan, A Coovadia, K D'Este, J Finn, et al. Cardiac arrest and cardiopulmonary resuscitation outcome reports: update and simplification of the utstein templates for resuscitation registries: a statement for healthcare professionals from a task force of the international liaison committee on resuscitation (american heart association, european resuscitation council, australian resuscitation council, new zealand resuscitation council, heart and stroke foundation of canada, inter-american heart foundation, resuscitation councils of southern africa) circulation. 2004; 110: 3385–3397. doi: 10.1161/01. *CIR*, 147236:15, 2014.

[32] Curtis N Sessler, Mark S Gosnell, Mary Jo Grap, Gretchen M Brophy, Pam V O'Neal, Kimberly A Keane, Eljim P Tesoro, and R K Elswick. The richmond agitation-sedation scale: validity and reliability in adult intensive care unit patients. *Am J Respir Crit Care Med*, 166(10):1338–44, Nov 2002.

[33] Neeraj Badjatia, Evangelia Strongilis, Errol Gordon, Mary Prescutti, Luis Fernandez, Andres Fernandez, Manuel Buitrago, J Michael Schmidt, Noeleen D Ostapkovich, and Stephan A Mayer. Metabolic impact of shivering during therapeutic temperature modulation: the bedside shivering assessment scale. *Stroke*, 39(12):3242–7, Dec 2008.

[34] Claudio Sandroni, Alain Cariou, Fabio Cavallaro, Tobias Cronberg, Hans Friberg, Cornelia Hoedemaekers, Janneke Horn, Jerry P Nolan, Andrea O Rossetti, and Jasmeet Soar. Prognostication in comatose survivors of cardiac arrest: an advisory statement from the european resuscitation council and the european society of intensive care medicine. *Resuscitation*, 85(12):1779–89, Dec 2014.

[35] L J Hirsch, S M LaRoche, N Gaspard, E Gerard, A Svoronos, S T Herman, R Mani, H Arif, N Jette, Y Minazad, J F Kerrigan, P Vespa, S Hantus, J Claassen, G B Young, E So, P W Kaplan, M R Nuwer, N B Fountain, and F W Drislane. American clinical neurophysiology

society's standardized critical care eeg terminology: 2012 version. *J Clin Neurophysiol*, 30(1):1–27, Feb 2013.

[36] Erik Westhall, Andrea O Rossetti, Anne-Fleur van Rootselaar, Troels Wesenberg Kjaer, Janneke Horn, Susann Ullén, Hans Friberg, Niklas Nielsen, Ingmar Rosén, Anders Åneman, David Erlinge, Yvan Gasche, Christian Hassager, Jan Hovdenes, Jesper Kjaergaard, Michael Kuiper, Tommaso Pellis, Pascal Stammet, Michael Wanscher, Jørn Wetterslev, Matt P Wise, Tobias Cronberg, and TTM-trial investigators. Standardized eeg interpretation accurately predicts prognosis after cardiac arrest. *Neurology*, 86(16):1482–90, Apr 2016.

[37] Derek C Angus, Jean-Paul Mira, and Jean-Louis Vincent. Improving clinical trials in the critically ill. *Crit Care Med*, 38(2):527–32, Feb 2010.

[38] Carolina A M Schurink, Christianne A Van Nieuwenhoven, Jan A Jacobs, Maja Rozenberg-Arska, Hans C A Joore, Erik Buskens, Andy I M Hoepelman, and Marc J M Bonten. Clinical pulmonary infection score for ventilator-associated pneumonia: accuracy and inter-observer variability. *Intensive Care Med*, 30(2):217–24, Feb 2004.

[39] Mervyn Singer, Clifford S Deutschman, Christopher Warren Seymour, Manu Shankar-Hari, Djillali Annane, Michael Bauer, Rinaldo Bellomo, Gordon R Bernard, Jean-Daniel Chiche, Craig M Coopersmith, Richard S Hotchkiss, Mitchell M Levy, John C Marshall, Greg S Martin, Steven M Opal, Gordon D Rubenfeld, Tom van der Poll, Jean-Louis Vincent, and Derek C Angus. The third international consensus definitions for sepsis and septic shock (sepsis-3). *JAMA*, 315(8):801–10, Feb 2016.

[40] GUSTO investigators. An international randomized trial comparing four thrombolytic strategies for acute myocardial infarction. *N Engl J Med*, 329(10):673–82, 09 1993.

[41] Patrick J Coppler, Jonathan Elmer, Luis Calderon, Alexa Sabedra, Ankur A Doshi, Clifton W Callaway, Jon C Rittenberger, Cameron Dezfulian, and Post Cardiac Arrest Service. Validation of the pittsburgh cardiac arrest category illness severity score. *Resuscitation*, 89:86–92, Apr 2015.

[42] Vivek N Iyer, Jayawant N Mandrekar, Richard D Danielson, Alexander Y Zubkov, Jennifer L Elmer, and Eelco F M Wijdicks. Validity of the four score coma scale in the medical intensive care unit. *Mayo Clin Proc*, 84(8):694–701, Aug 2009.

A The FOUR SCORE

**Eye response**	
Eyelids open and tracking, or blinking on command	4
Eyelids open but not tracking	3
Eyelids closed but open to loud voice	2
Eyelids closed but open to pain	1
Eyelids closed with pain	0
**Motor response**	
Makes sign (thumbs-up, fist, other)	4
Localising to pain	3
Flexion response to pain	2
Extension response to pain	1
No response to pain	0
Generalised myoclonic status	0
**Brainstem response**	
Pupil reflexes present, corneal reflexes present and cough present	4
One pupil wide and fixed, corneal reflexes present and cough present	3
Pupil reflexes absent, corneal reflexes present	2
Pupil reflexes present, corneal reflexes absent	2
Pupil reflexes absent, corneal reflexes absent, cough present	1
Pupil reflexes absent, corneal reflexes absent, cough absent	0
**Breathing**	
Not intubated with regular breathing	4
Not intubated with Cheyne- Stokes type of breathing	3
Not intubated with irregular breathing	2
Not intubated with apnea	0
Intubated with breathing above ventilator rate	1
Intubated with breathing at ventilator rate	0

In contrast to previous trials on cardiac arrest, the TTM2-trial will not use the Glasgow Coma Score in any reporting. There are several reasons for this:

- The FOUR-score offers a less equivocal inclusion criteria as a "fist" or "thumbs-up" response is required for a motor score of 4. This also applies to the definition of awakening which is made clearer by requiring a limb movement rather than only eye movements
- The FOUR-score can be rated in the intubated patient and (as part of the Pittsburgh cardiac arrest category [41, 42]

Links:
Figure of FOUR-score from Iyer et.al [42]
FOUR-score calculator

B The modified Rankin scale (mRS)

Score	Description
**0**	No symptoms at all
**1**	No significant disability despite symptoms; able to carry out all usual duties and activities
**2**	Slight disability; unable to carry out all previous activities , but able to look after own affairs without assistance
**3**	Moderate disability; requiring some help, but able to walk without assistance
**4**	Moderate severe disability; unable to walk without assistance and unable to attend to own bodily needs without assistance
**5**	Severe disability; bedridden, incontinent and requiring constant nursing care and attention
**6**	Death

C The Glasgow outcome scale - extended (GOSE)

Score	Description
**1**	Death
**2**	Vegetative state
**3**	Lower severe disability
**4**	Upper severe disability
**5**	Lower moderate disability
**6**	Upper moderate disability
**7**	Lower good recovery
**8**	Upper good recovery

D Charter for the DSMC of the TTM2-trial

Clinical Trial no. NCT02908308

D.1 Introduction

The Charter will define the primary responsibilities of the DSMC, its relationship with other trial components, its membership, and the purpose and timing of its meetings. The Charter will also provide the procedures for ensuring confidentiality and proper communication, the statistical monitoring guidelines to be implemented by the DSMC, and an outline of the content of the Open and Closed Reports that will be provided to the DSMC.

D.2 Primary responsibilities of the DSMC

The DSMC will be responsible for safeguarding the interests of trial participants, assessing the safety and efficacy of the interventions during the trial, and for monitoring the overall conduct of the clinical trial. The DSMC will provide recommendations about stopping or continuing the trial to the Steering Group (SG) of the TTM2-trial. To contribute to enhancing the integrity of the trial, the DSMC may also formulate recommendations relating to the selection/recruitment/retention of participants, their management, improving adherence to protocol-specified regimens and retention of participants, and the procedures for data management and quality control. The DSMC will be advisory to the SG. The SG will be responsible for promptly reviewing the DSMC recommendations, to decide whether to continue or terminate the trial, and to determine whether amendments to the protocol or changes in trial conduct are required.

The DSMC is planned by protocol to meet physically in order to evaluate the planned interim analysis of the TTM2-trial. The interim analyses will be performed by an independent statistician selected by the member of the DSMC. The DSMC may additionally meet whenever they decide, contact each other by telephone or e-mail in order to discuss the safety for trial participants. The Principal investigator has the responsibility to report monthly to the DSMC the overall number of Serious Adverse Events (SAE). The DSMC can request at any time during the trial the distribution of events, including outcome measures and SAEs, according to intervention groups. The recommendations of the DSMC regarding stopping, continuing or changing the design of the trial should be communicated without delay to the SG of the TTM2-trial. The SG has the responsibility to inform as fast as possible, and no later than 48 hrs, all investigators of the trial and the departments including patients in the trial the recommendation of the DSMC and the SG decision hereof.

D.3 Members of the DSMC

The DSMC is an independent multidisciplinary group consisting of clinicians and a biostatistician that, collectively, has experience in the management of ICU patients and in the conduct, monitoring and analysis of randomised clinical trials.

D.3.1 Members of the DSMC-committee

**Kathy Rowan**	Intensive Care National Audit & Research Centre, UK (Chair)
**David Harrison**	Intensive Care National Audit & Research Centre, UK
**Paul Mouncey**	Intensive Care National Audit & Research Centre, UK
**Duncan Young**	Nuffield Department of Clinical Neurosciences, University of Oxford, UK
**Manu Shankar-Hari**	Guy's and St Thomas's NHS Foundation Trust, London, UK

D.4 Conflicts of interest

DSMC membership has been restricted to individuals free of conflicts of interest. The source of these conflicts may be financial, scientific, or regulatory in nature. Any DSMC members who develop significant conflicts of interest during the course of the trial should resign from the DSMC. DSMC membership is to be for the duration of the clinical trial. If any members leave the DSMC during the course of the trial, the SG will appoint the replacement(s).

D.5 Formal interim analysis meeting

One 'Formal Interim Analyses' meetings will be held to review data relating to treatment efficacy, patient safety, and quality of trial conduct. The three members of the DSMC will meet when 180-day follow-up data of 600 patients have been obtained.

D.6 Proper communication

To enhance the integrity and credibility of the trial, procedures will be implemented to ensure the DSMC has sole access to evolving information from the clinical trial regarding comparative results of efficacy and safety data, aggregated by treatment group (0,1). An exception will be made to permit access to an independent statistician who will be responsible for serving as a liaison between the database and the DSMC. At the same time, procedures will be implemented to ensure that proper communication is achieved between the DSMC and the trial investigators. To provide a forum for exchange of information among various parties who share responsibility for the successful conduct of the trial, a format for Open Sessions and Closed Sessions will be implemented. The intent of this format is to enable the DSMC to preserve confidentiality of the comparative efficacy results while at the same time providing opportunities for interaction between the DSMC and others who have valuable insights into trial-related issues.

D.7 Closed Sessions

Sessions involving only DSMC members and generate the Closed Reports will be held to allow discussion of confidential data from the clinical trial, including information about the relative efficacy and safety of interventions. In order to ensure that the DSMC will be fully informed in its primary mission of safeguarding the interest of participating patients, the DSMC will be blinded in its assessment of safety and efficacy data. However, the DSMC can request unblinding from the SG.

D.8 Open Reports

For each DSMC meeting, Open Reports will be provided available to all who attend the DSMC meeting. The Reports will include data on recruitment and baseline characteristics, and pooled data on eligibility violations, completeness of follow-up, and compliance. The primary trial statistician will prepare these Open Reports. Closed Reports will include analysis of the primary efficacy outcome measure. In addition, analyses of the secondary outcome measures and serious adverse events will also be reported. These Closed Reports will be prepared by an independent biostatistician, with assistance from the trial biostatisticians, in a manner that allow them to remain blinded. The Closed Reports should provide information that is accurate, with follow-up on mortality that is complete to within two months of the date of the DSMC meeting. The Reports should be provided to DSMC members approximately three days prior to the date of the meeting.

D.9 Minutes of the DSMC Meetings

The DSMC will prepare minutes of their meetings. The Closed Minutes will describe the proceedings from all sessions of the DSMC meeting, including the listing of recommendations by the Committee. Because it is likely that these minutes may contain unblinded information, it is important that they are not made available to anyone outside the DSMC.

D.10 Recommendations to the Steering Committee

After the interim analysis meeting, the DSMC will make a recommendation to the SC to continue, hold or terminate the trial. This recommendation will be based primarily on safety and efficacy considerations and will be guided by statistical monitoring guidelines defined in this Charter and the trial protocol. The SC is jointly responsible with the DSMC for safeguarding the interests of participating patients and for the conduct of the trial. Recommendations to amend the protocol or conduct of the trial made by the DSMC will be considered and accepted or rejected by the SC. The SC will be responsible for deciding whether to continue, hold or stop the trial based on the DSMC recommendations. The DSMC will be notified of all changes to the trial protocol or conduct. The DSMC concurrence will be sought on all substantive recommendations or changes to the protocol or trial conduct prior to their implementation.

D.11 Statistical monitoring guidelines

The outcome parameters are defined in the TTM2-trial protocol. For the two intervention groups, the DSMC will evaluate data on:

- The primary outcome measure - all cause mortality at 180 days
- The secondary outcome measures - The composite outcome of all cause mortality and poor functional outcome (mRS 4 to 6) at 30 days and at 180 days.
- Serious adverse events - SAEs

The DSMC will be provided with these data from the Coordinating Centre as:

- a. Number of patients randomised

- b. Number of patients randomised per intervention group (0,1)
- c. Number of patients stratified per. stratification variable per intervention group (0,1)
- d. Number of events, according to the outcomes, in the two groups

Based on evaluations of these outcomes, the DSMC will decide if they want further data from the Coordinating Centre and when next to perform analyses of the data. For analyses, the data will be provided in one file as described below. Based on the analyses of the primary outcome measure and SAEs, the DSMC will use P<0.001 as the statistical limit to guide its recommendations regarding early termination of the trial, at the first formal meeting. Based on the 30 day composite outcome analysis, the DSMC will use P<0.001 Lan-DeMets group sequential monitoring boundaries as the statistical limit to guide its recommendations regarding early termination of the trial. DSMC should also be informed about all unexpected SAEs occurring in the two groups of the trial.

The DSMC may also be asked to ensure that procedures are properly implemented to adjust trial sample size or duration of follow-up to restore power, if protocol specified event rates are inaccurate. If so, the algorithm for doing this should be clearly specified.

D.12 Conditions for transfer of data from the Coordinating Centre to the DSMC

The DSMC shall be provided with the data described below in one file The DSMC will be provided with an Excel database containing the data defined as follows:

- Row 1 contains the names of the variables (to be defined below).
- Row 2 to N (where N-1 is the number of patients who have entered the trial) each contains the data of one patient.
- Column 1 to p (where p is the number of variables to be defined below) each contains in row 1 the name of a variable and in the next N rows the values of this variable.

The values of the following variables should be included in the database:

A. **PtID:** a number that uniquely identifies the patient.

B. **Rdcode:** The randomisation code (group 0 or 1). The DSMC is not to be informed on what intervention the groups received.

C. **1.EndInd:** Primary outcome measure indicator (1 if patient fulfilled the primary outcome measure at day 180 and 0 if the patient did not).

D. **180MInd:** 180 day-mortality indicator (2 if patient is censored, 1 if patient was dead, and 0 if the patient was alive at day 90).

E. **180MBNlnd:** Mortality and poor functional outcome at 180 days (2 if patient is censored, 1 if patient fulfils criteria, and 0 if the patient does not).

F. **30MBNlnd:** Mortality and poor functional outcome at hospital discharge (2 if patient is censored, 1 if patient fulfils criteria, and 0 if the patient does not).

G. **SAEInd:** Serious Adverse Event indicator (1 if patient has had a SAE during ICU stay and 0 if the patient did not).

E Version History

Version 1.0 - published online and signed May 17, 2017

Version 1.1 - published online and signed November, 2017

- General. Typing errors corrected
- Title page, Changed title of Dr. Niklas Nielsen to Chief Investigator.
- 2.3. Removed section included in parentheses of "to a low target level (32-33°C)" to clarify that 33°C is the correct target temperature.
- 3.2 Clarification of outcome "Days alive outside hospital". Description now states "days alive after first hospital discharge within 180 days".
- 4.1 Clarification of initial unconsciousness by adding "no verbal response to pain".
- 5.2 removed "cooled with a device to <33°C". Clarification that target temperature is 33°C as lower temperatures might not be possible.
- 5.5.7 Layout of criteria changed.
- 7.7 First paragraph rephrased. Clarification that initial temperature target is 33°, but that an initial device setting of 32°is allowed. Second paragraph added description of recommended device (commercially available and closed-loop).
- 8 and 10 eCRF provider changed from Lytics to Spiral.
- 8.1.1 Removed "national identification number" from data collection.
- 8.1.3 Added "Previous known cardiomyopathy" to data collection
- 8.10. Changed reference point of blood samples from time after arrest, to time after randomisation for consistency. Changed name of "admission-samples" to "0h-samples".
- 11.1. Added references for GUSTO-trial and Sepsis 3.
- 11.1 Data added: Pneumonia (modified CPIS)
- 12.1 Change of power calculation after reexamination of registry data - higher mortality expected, from 45% to 51.25%. Same absolute risk reduction of 7.5%.
- 12.5.1 Added names of members' in the DSMC
