# Supplementary material for: Long-Term Outcomes and Recovery Trajectories in Out-of-Hospital Cardiac Arrest: A 2-Year Follow-Up of the Randomized Clinical TTM2 Trial
Source: JAMA Neurol. 2026 Feb 16;83(4):339–47. doi: 10.1001/jamaneurol.2025.5614 (PMC12910457; doi:10.1001/jamaneurol.2025.5614)
Supplement: Supplement 3. — Data Sharing Statement [file jamaneurol-e255614-s003.pdf]

Data Sharing Statement

Hultgren. Long-Term Outcomes and Recovery Trajectories in Out-of-Hospital Cardiac Arrest.
JAMA Neurol. Published February 16, 2026. doi:10.1001/jamaneurol.2025.5614

Data
Additional Information: ClinicalTrials.gov, identifier: NCT02908308.
Data available: Yes
Data types: Deidentified participant data
How to access data: Data will be made available upon reasonable request.
When available: With publication

Supporting Documents
Document types: None

Additional Information
Who can access the data: N/A
Types of analyses: N/A
Mechanisms of data availability: N/A
